# Supplementary figures and images for: ORANGE: A CRISPR/Cas9-based genome editing toolbox for epitope tagging of endogenous proteins in neurons
Source: PLoS Biol. 2020 Apr 10;18(4):e3000665. doi: 10.1371/journal.pbio.3000665 (PMC7176289; doi:10.1371/journal.pbio.3000665)

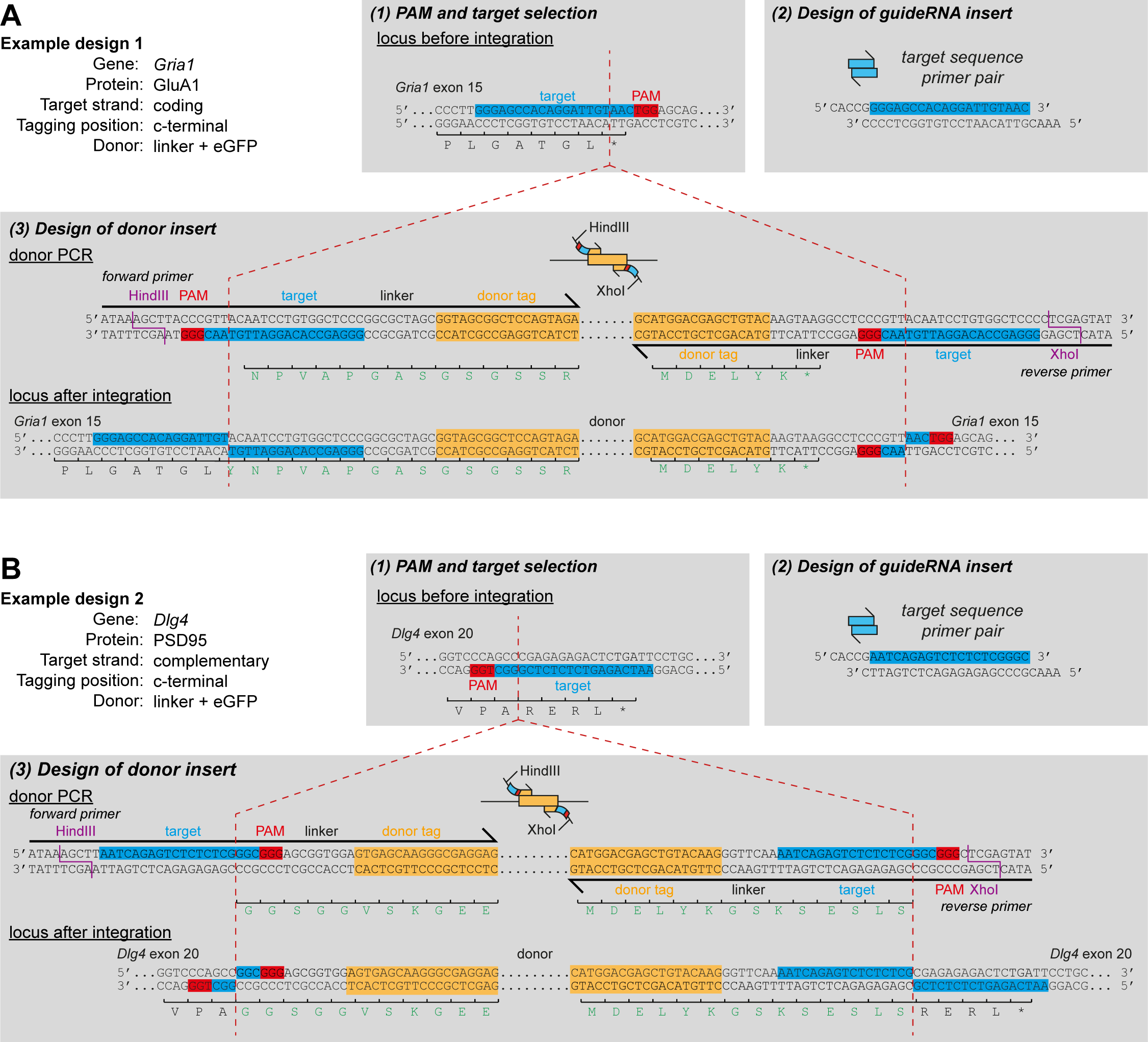

Supplement: S1 Fig — (A and B) Examples of knock-in construct design for Gria1 (A) and Dlg4 (B), which contain target sequences in opposite genomic strands. The target sequence is indicated in blue, the PAM sequence is in red, and the part of the primer used for PCR amplification of the donor DNA is shown in yellow. Amino acid sequence is shown under the sequences. Asterisk indicates stop codon. Red dotted lines indicate position of Cas9 cleavage and sites of integration. Purple line indicates restriction enzyme sites used for cloning into pORANGE. Dlg4, Discs Large MAGUK Scaffold Protein 4; Gria1, glutamate ionotropic receptor AMPA type subunit 1; ORANGE, Open Resource for the Application of Neuronal Genome Editing; PAM, protospacer adjacent motif. (TIF) [file pbio.3000665.s001.tif]

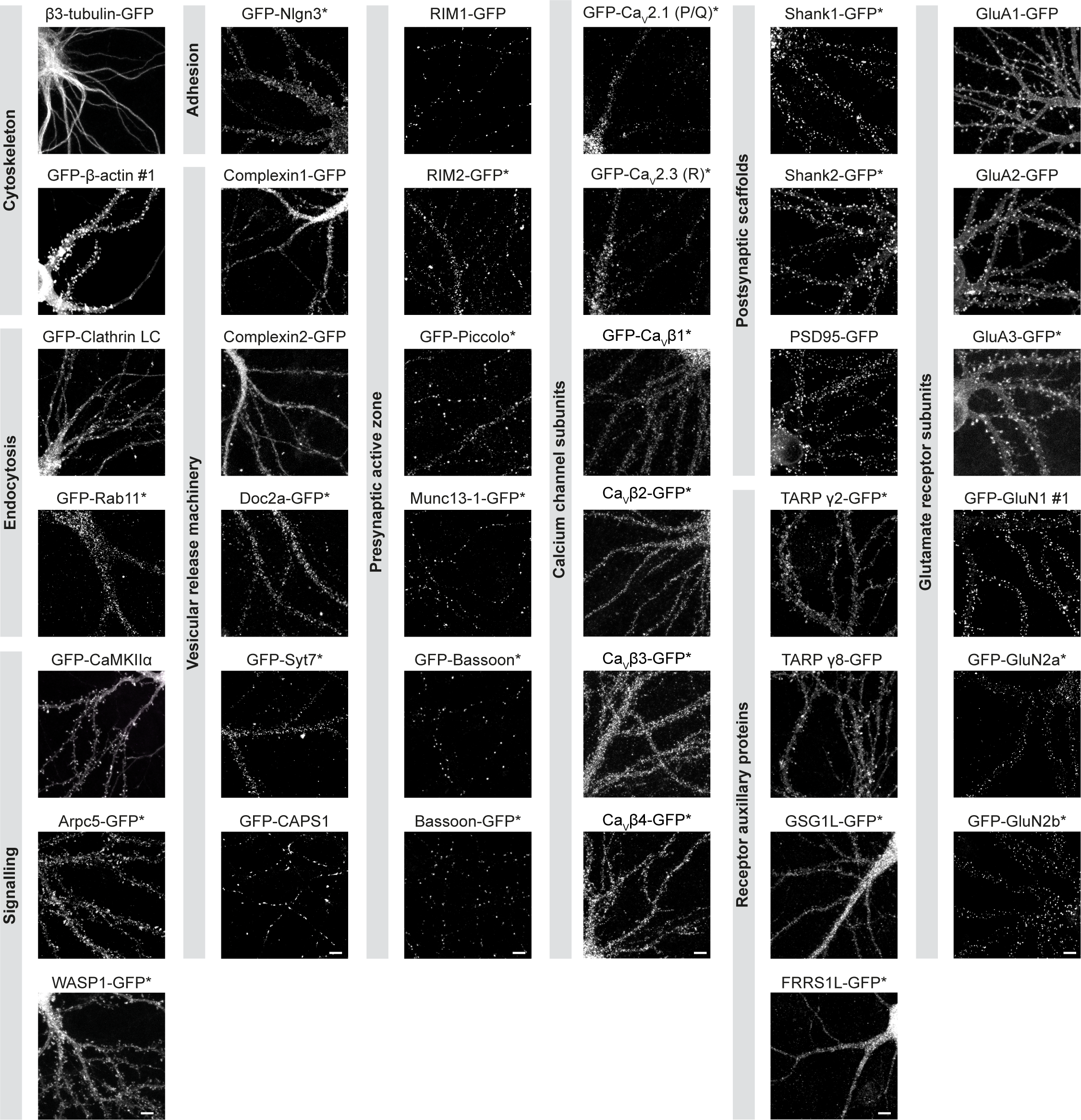

Supplement: S2 Fig — Representative images of cultured hippocampal knock-in neurons. Examples shown are used for zooms shown in Fig 2D. DIV 21. Asterisk indicates signal enhanced using anti-GFP antibodies (Alexa488 or Alexa647). Scale bar, 5 μm. GFP, green fluorescent protein; ORANGE, Open Resource for the Application of Neuronal Genome Editing. (TIF) [file pbio.3000665.s002.tif]

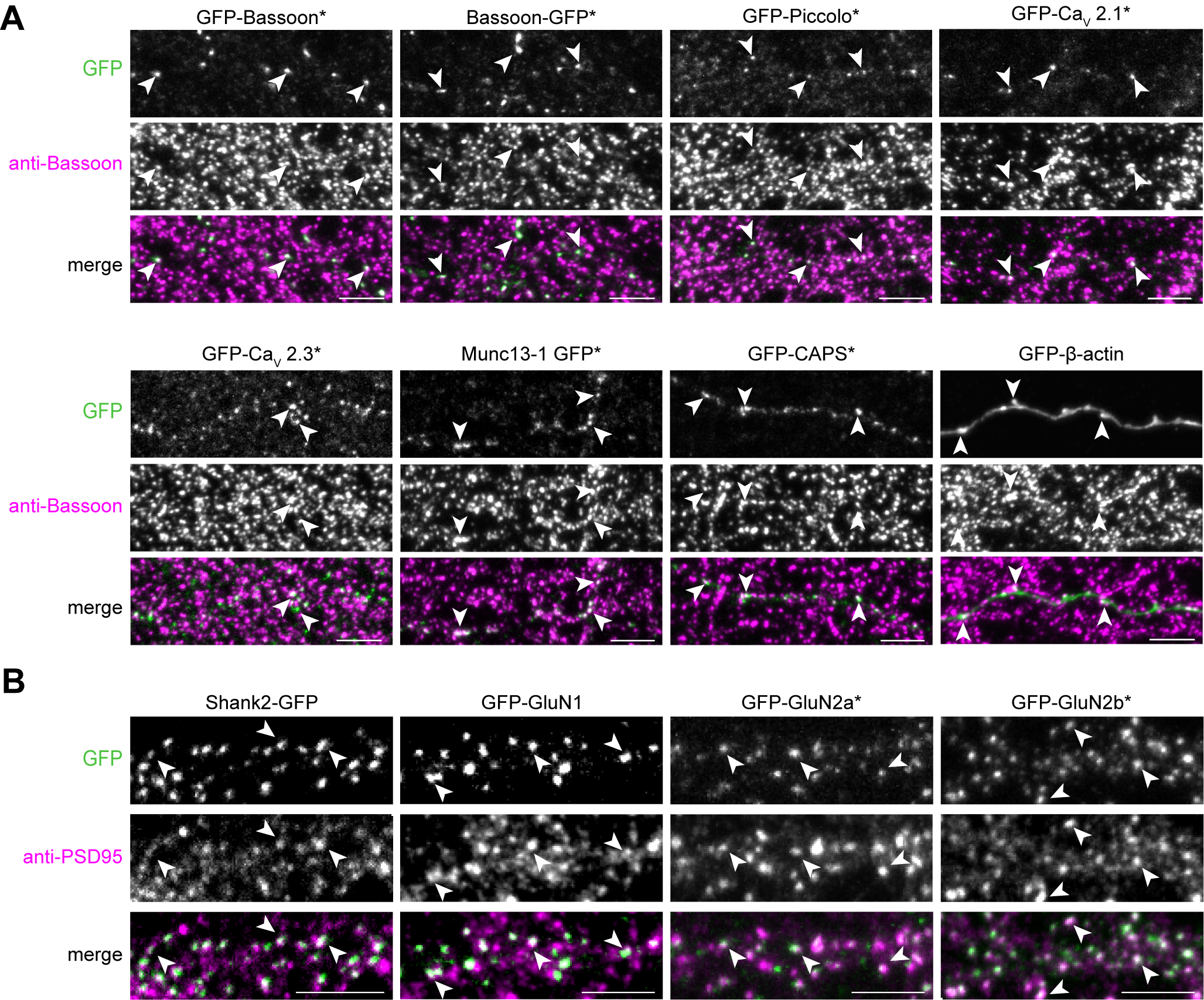

Supplement: S3 Fig — (A) Examples of GFP knock-in (green) relative to anti-Bassoon staining (magenta, Alexa647) as presynaptic marker or (B) anti-PSD95 staining (magenta, Alexa647) as postsynaptic marker in cultured hippocampal neurons. Asterisk indicates signal enhancement using anti-GFP antibodies (Alexa488). Scale bars, 5 μm. Arrows indicate examples of GFP-positive objects. GFP, green fluorescent protein; ORANGE, Open Resource for the Application of Neuronal Genome Editing. (TIF) [file pbio.3000665.s003.tif]

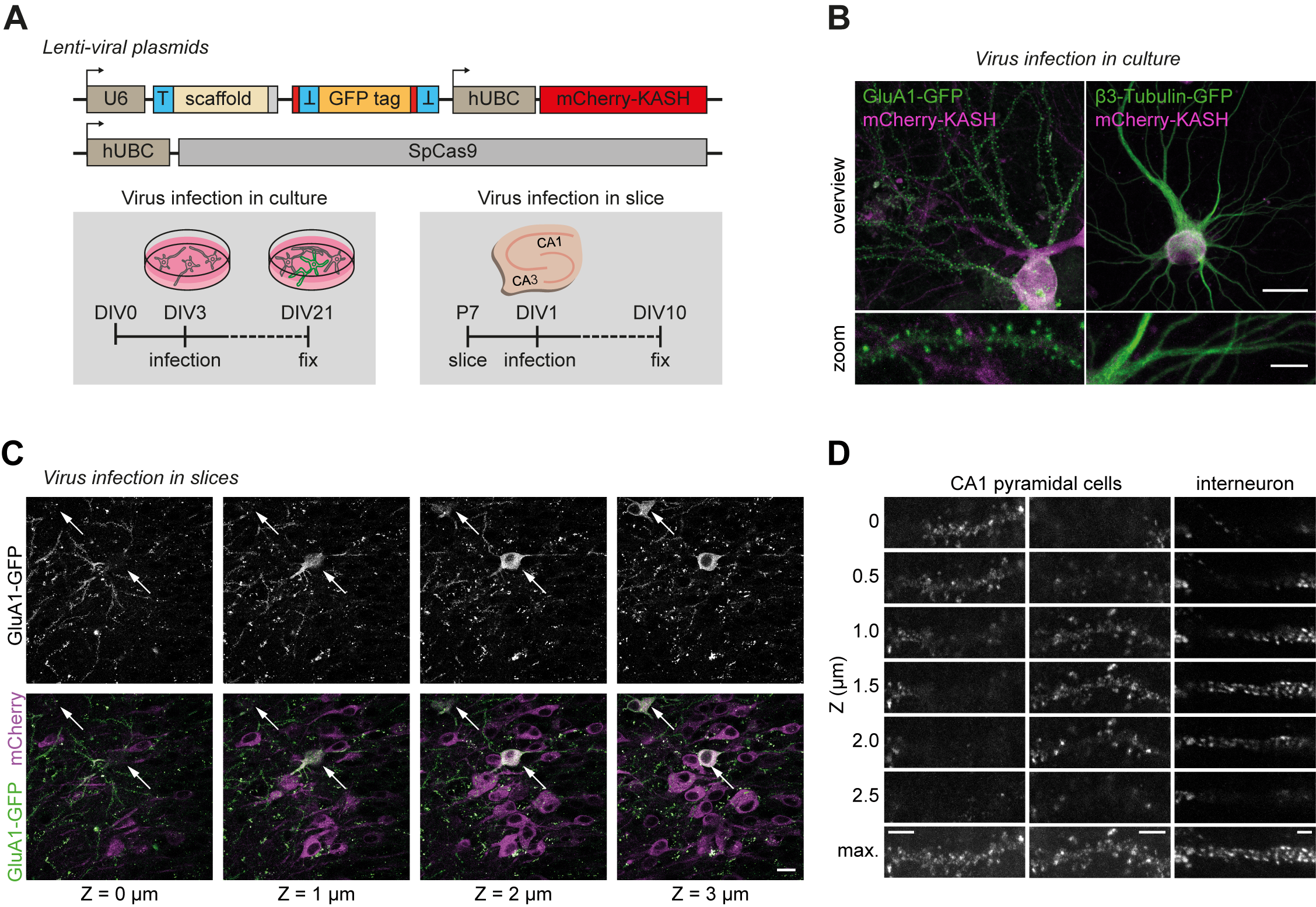

Supplement: S4 Fig — (A) Overview of lentiviral constructs and timeline showing age of infection and fixation. (B) Representative images of infected (magenta) primary rat hippocampal neurons positive for GluA1-GFP knock-in or β3-tubulin-GFP knock-in (green). Scale bars, 20 μm and 5 μm for the overview and zooms, respectively. (C) Representative images of GluA1-GFP knock-in in organotypic hippocampal slices from mice. Shown are a series of individual 1-μm planes from a Z-stack. Arrows indicate GFP-positive cells. Scale bar, 20 μm. (D) Representative zooms of GluA1-GFP knock-in dendrites from a CA1 pyramidal cell and an aspiny interneuron. Shown are individual 0.5-μm planes from a Z-stack and the maximum projection (max). Scale bar, 2 μm. CA1, cornu ammonis region 1; GFP, green fluorescent protein; GluA1, Glutamate receptor AMPA 1; ORANGE, Open Resource for the Application of Neuronal Genome Editing. (TIF) [file pbio.3000665.s004.tif]

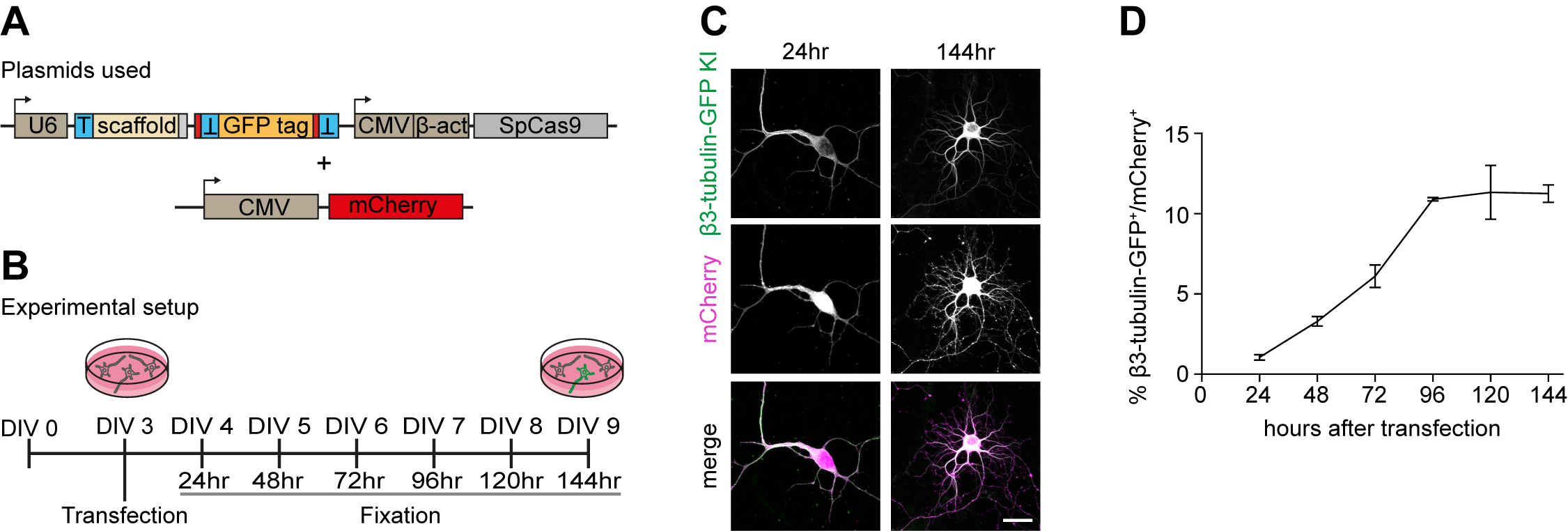

Supplement: S5 Fig — (A) Schematic overview of knock-in and mCherry reporter plasmids and (B) experimental setup. (C) Representative images of β3-tubulin-GFP knock-in (green) cotransfected with an mCherry fill (magenta) fixed 24 hours (DIV 4) and 144 hours (DIV 9) after transfection. Scale bar, 20 μm. (D) Quantification of β3-tubulin-GFP knock-in efficiency over time as percentage of transfected (mCherry-positive) neurons. Data are represented as means ± SEM. Underlying data can be found in S1 Data. DIV, day in vitro; GFP, green fluorescent protein; ORANGE, Open Resource for the Application of Neuronal Genome Editing. (TIF) [file pbio.3000665.s005.tif]

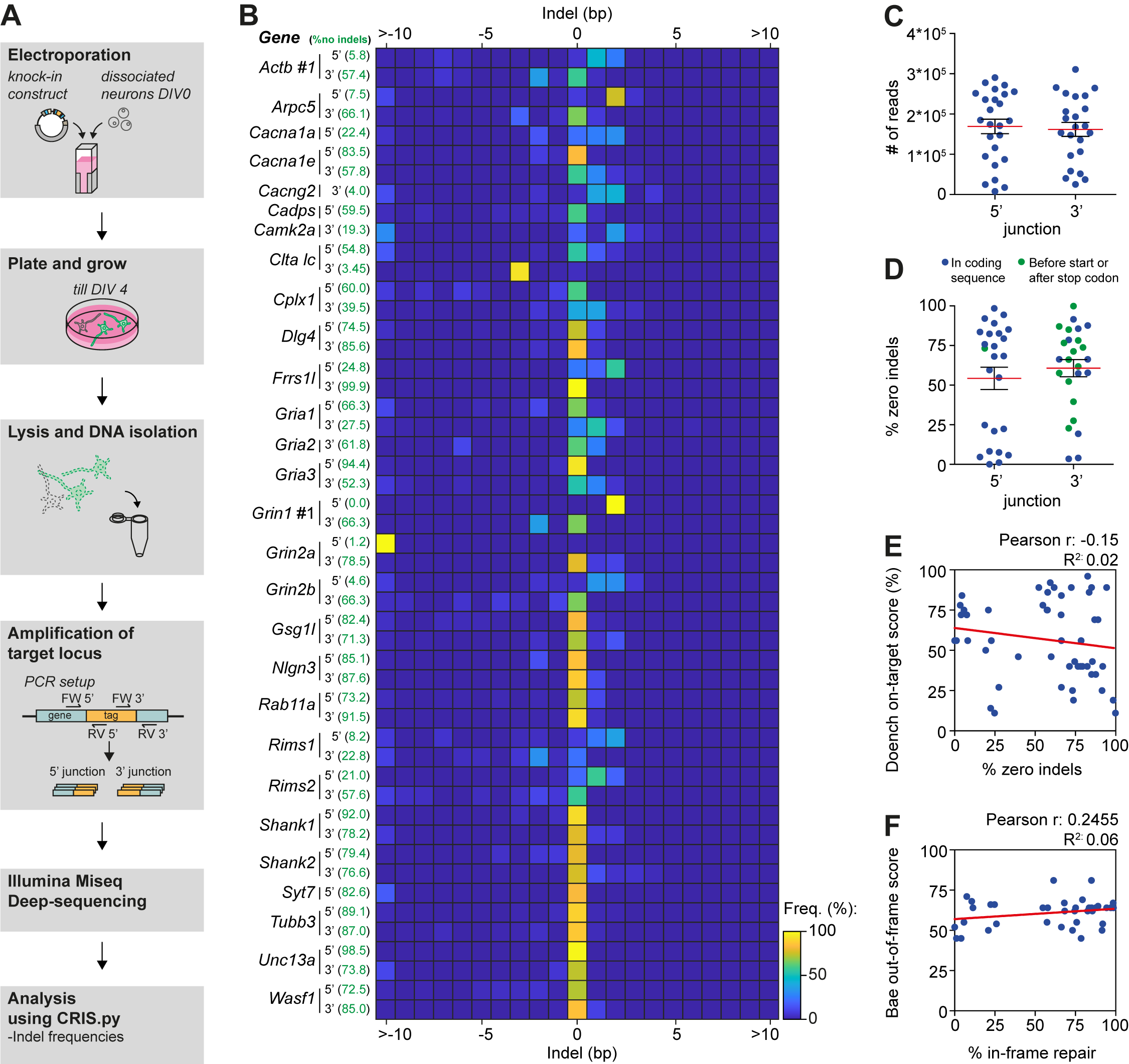

Supplement: S6 Fig — (A) Schematic overview of experimental setup. Neurons were electroporated immediately after dissociation and cultured until DIV 4. Genomic DNA was isolated, and the 5′ and 3′ junctions of integration were amplified with PCR, pooled, and subjected to next-generation sequencing. (B) Heatmap summarizing the sequencing results for 5′ and 3′ junction amplicons of the indicated knock-ins. Heatmap is color-coded for the frequency of indel size, as analyzed using CRIS.py. For a few genes, we were only able to amplify one of the two junctions with PCR. (C) Average number of reads obtained with deep sequencing for all successfully analyzed knock-ins (mean 5′: 1.69 × 10^5^ reads ± 0.18 × 10^5^, 3′: 1.57 × 10^5^ ± 0.16 × 10^5^). (D) Accuracy of knock-in plotted for each junction. Plotted points indicate percentage of zero indels from all knock-ins in (B) (mean 5′: 54.2% ± 7.0%, 3′: 60.7% ± 5.4%). Green points indicate minor mutations that do not influence the reading frame for this particular integration (e.g., frame shift after stop codon). (E) Correlation graph between zero indel frequency per amplicon and Doench on-target score of the gRNA target sequence. (F) Correlation graph between correct reading frame integration frequency and Bae out-of-frame score of the gRNA target sequence. Data are presented as means ± SEM. Underlying data can be found in S1 Data. DIV, day in vitro; gRNA, guide RNA. (TIF) [file pbio.3000665.s006.tif]

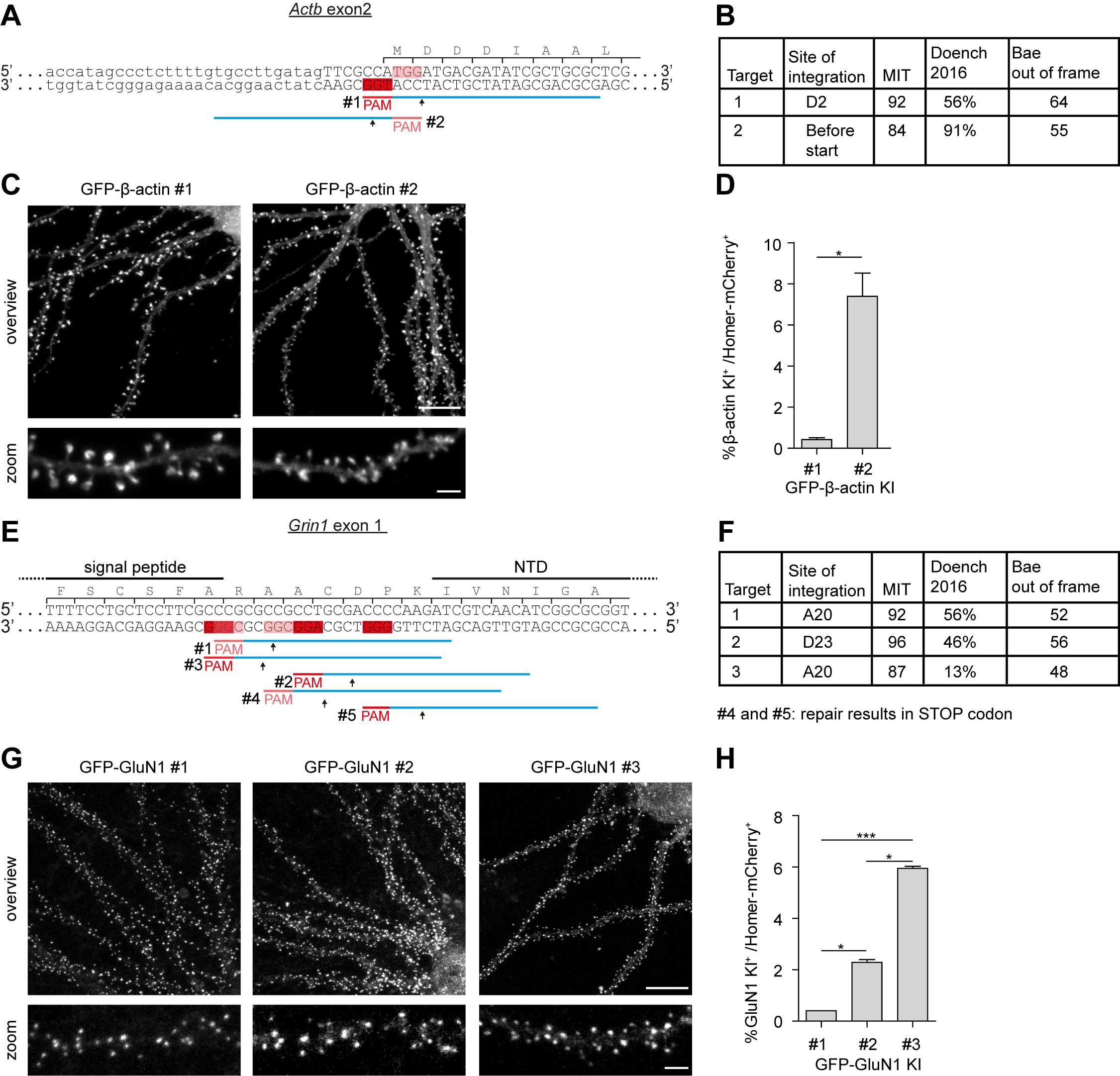

Supplement: S7 Fig — (A and E) Genomic regions of the Actb and Grin1 genes around the targeted integration site are shown. The PAM (red line and red shaded boxes) and target sequences (blue) are shown below for each of the tested knock-ins. Intron sequences are in lowercase, and exon sequences are in uppercase. Additional protein information is shown above the sequence. (B and F) Tables containing information about the site of integration at the protein level and MIT, Doench, and Bae scores of the individual guide RNA sequences (determined based on rat genomic sequence from the UCSC RGSC5.0/rn5 genome assembly). (C and G) Representative images of neurons transfected with the various β-actin (C) or GluN1 (G) knock-in constructs targeting the PAM sites shown in (A) and (E), respectively. Scale bars, 10 μm and 2 μm for the overviews and zooms, respectively. (D and H) Knock-in efficiency determined as the percentage of GFP-β-actin (D) or GFP-GluN1 (H)-positive neurons coexpressing Homer1c-mCherry. Data are presented as means ± SEM. * P < 0.05, *** P < 0.001, ANOVA or Student t test. Underlying data can be found in S1 Data. Actb, Actin Beta; GFP, green fluorescent protein; GluN1, Glutamate receptor NMDA 1; Grin1, glutamate ionotropic receptor NMDA type subunit 1; PAM, protospacer adjacent motif; UCSC, University of California Santa Cruz. (TIF) [file pbio.3000665.s007.tif]

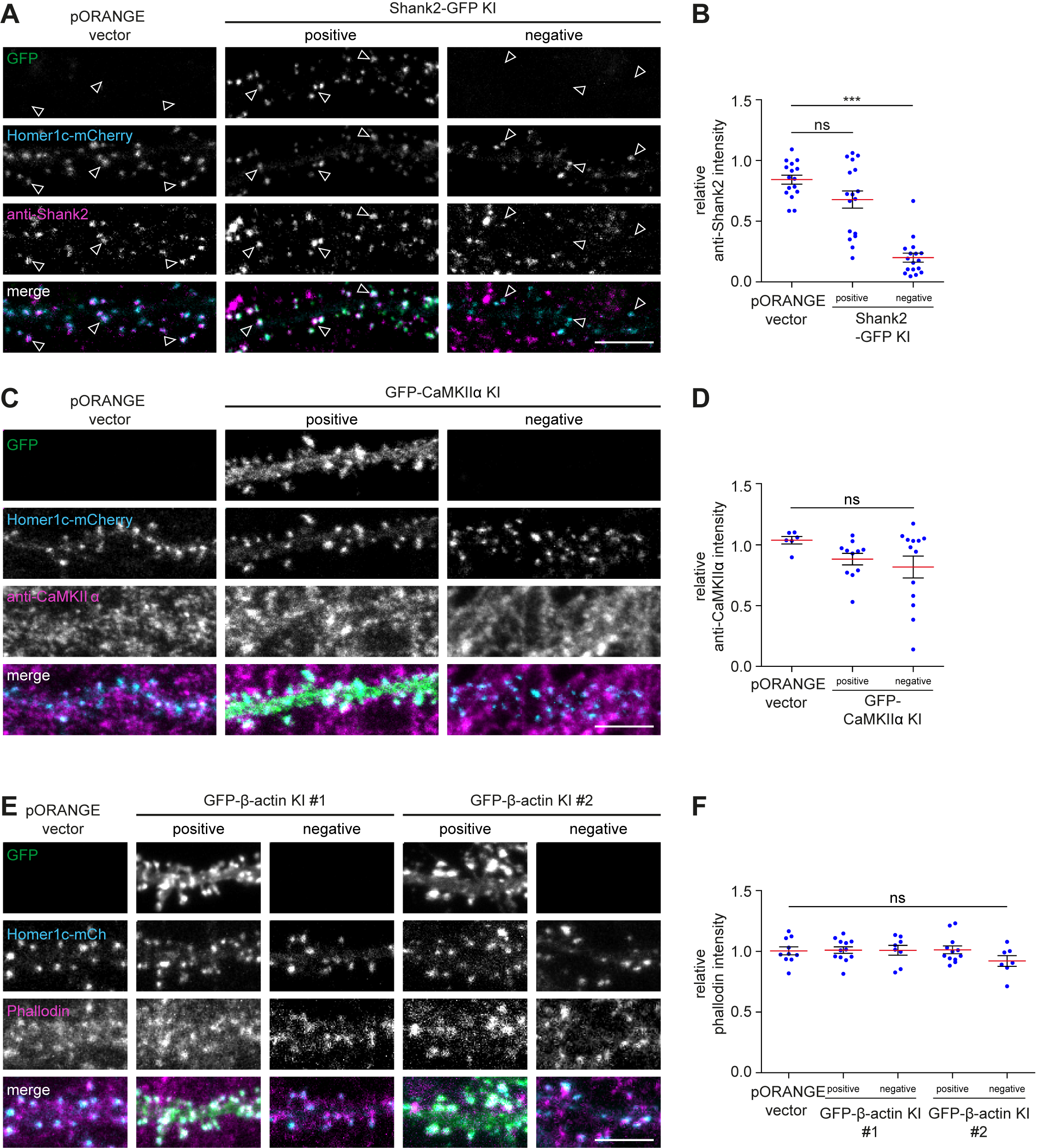

Supplement: S8 Fig — (A) Representative images of neurons transfected with Homer1c-mCherry (cyan) together with pORANGE empty vector as control or Shank2-GFP knock-in (green). Neurons were stained with anti-Shank2 (magenta, Alexa647). (C) Images of neurons transfected with Homer1c-mCherry (cyan) together with pORANGE empty vector as control GFP-CaMKIIα knock-in (green). Neurons were stained with anti-CaMKIIα (magenta, Alexa647). (E) Neurons transfected with Homer1c-mCherry together with pORANGE empty vector as control, GFP-β-actin knock-in #1 or GFP-β-actin knock-in #2 (green). Neurons were stained for F-actin using phalloidin (magenta, Alexa647). Scale bar, 5 μm. (B, D, and F) Quantification of protein levels relative to transfected neurons. (B) Relative fluorescence intensity: control: 0.84 ± 0.04, n = 16 neurons, Shank2-GFP knock-in: 0.67 ± 0.07, n = 17 neurons, P > 0.05, knock-in negative: 0.20 ± 0.04, n = 17 neurons, P < 0.01, ANOVA. (D) Control: 1.04 ± 0.03, n = 6 neurons, GFP-CaMKIIα knock-in: 0.88 ± 0.05, n = 11 neurons, P > 0.05, knock-in negative: 0.82 ± 0.09, n = 13 neurons, P > 0.05, ANOVA. (F) Control: 1.00 ± 0.03, n = 10 neurons, GFP-β-actin knock-in #1: 1.01 ± 0.03, n = 12 neurons, P > 0.05, knock-in #1 negative: 1.01 ± 0.04, n = 8 neurons, P > 0.05, GFP-β-actin knock-in #2: 1.01 ± 0.03, n = 12 neurons, P > 0.05, knock-in #2 negative: 0.92 ± 0.04, n = 7 neurons, P > 0.05, ANOVA. Data are presented as means ± SEM. ***P < 0.001, ANOVA. Underlying data can be found in S1 Data. CaMKIIα, Calcium/calmodulin-dependent protein kinase type II subunit alpha; GFP, green fluorescent protein; ns, not significant; ORANGE, Open Resource for the Application of Neuronal Genome Editing; SHANK2, SH3 and multiple ankyrin repeat domains protein 2. (TIF) [file pbio.3000665.s008.tif]

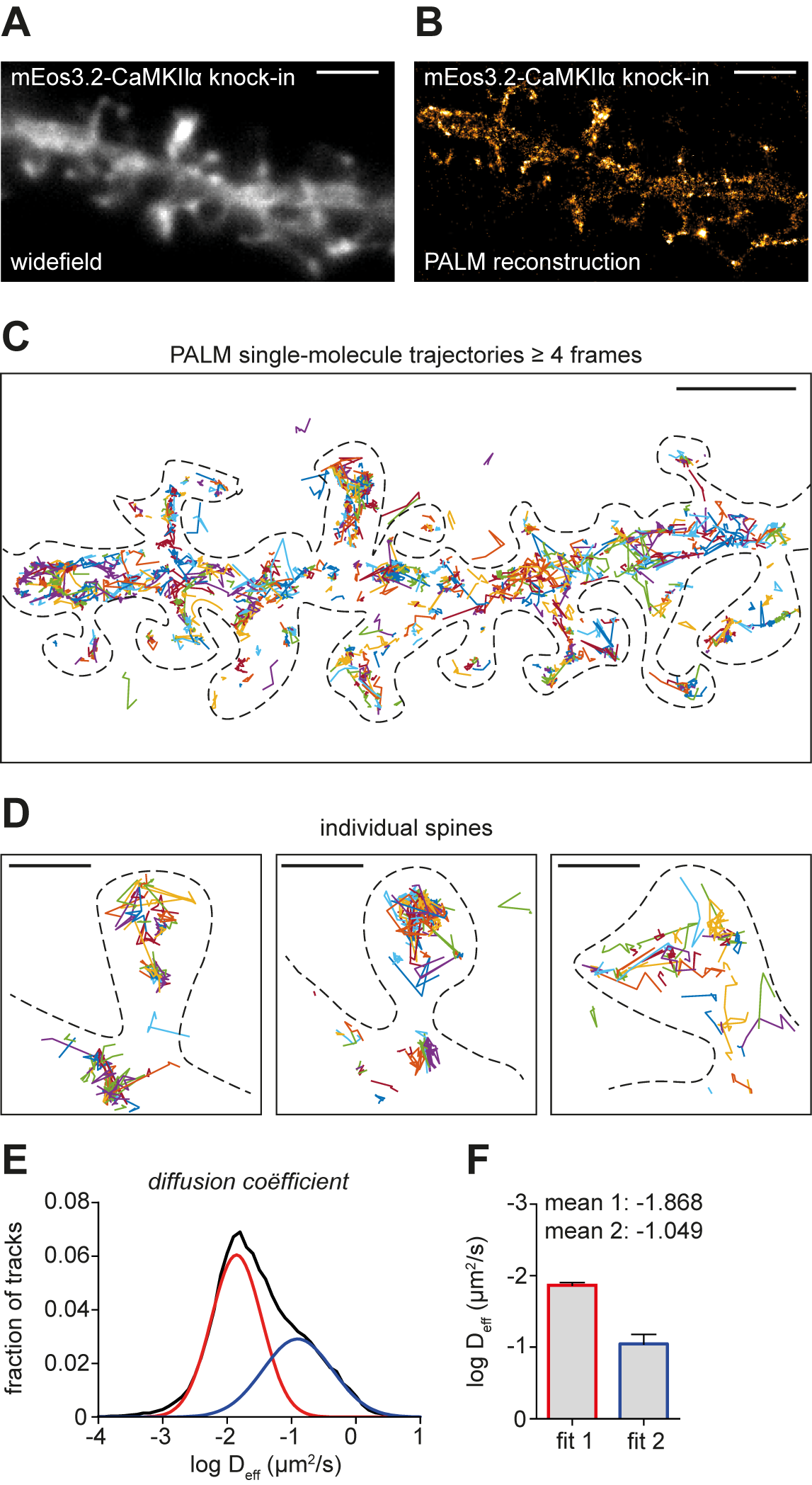

Supplement: S9 Fig — (A) Example of a dendrite expressing mEos3.2-CaMKIIα knock-in. Scale bar, 2 μm. (B) Single-molecule PALM reconstruction of dendrite shown in (A). Scale bar, 2 μm. (C) Individual single-molecule trajectories. Scale bar, 2 μm. Dotted line indicates cell outline. (D) Representative zooms of single-molecule trajectories in individual spines. Scale bar, 200 nm. (E) Frequency distribution of diffusion coefficients derived from single-molecule trajectories (black line). Mixed Gaussian fits (red and blue) indicate two kinetic populations. (F) Quantification of mean diffusion coefficient for each of the two kinetic populations. Data are presented as means ± SEM. Underlying data can be found in S1 Data. CaMKIIα, Calcium/calmodulin-dependent protein kinase type II subunit alpha; mEos3.2, monomeric Eos 3.2; PALM, photoactivated localization microscopy. (TIF) [file pbio.3000665.s009.tif]

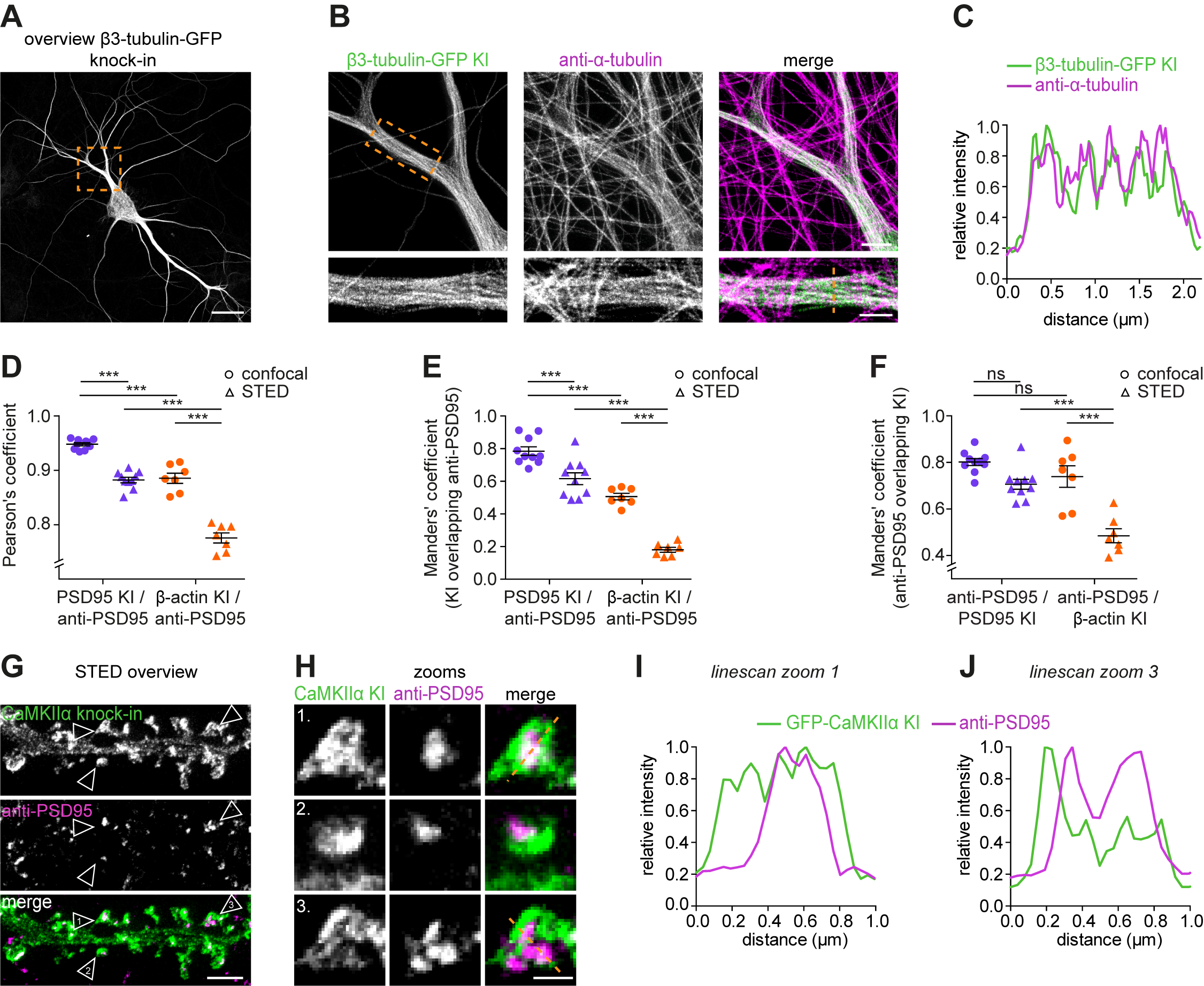

Supplement: S10 Fig — (A) Representative gSTED overview image and zooms (B) of β3-tubulin-GFP knock-in neurons (DIV 7) extracted and stained with anti-GFP (green, ATTO647N) and anti-α-tubulin (magenta, Alexa594). Scale bars, 20 μm, 4 μm, and 2 μm for the overview and zooms, respectively. (C) Intensity profile along the line indicated in (B). (D) PCC quantifying colocalization between PSD95-GFP or GFP-β-actin knock-in signal with anti-PSD95 staining intensity. Related to Fig 6F–6K. (E and F) Manders’ correlation of PSD95-GFP or GFP-β-actin knock-in overlapping with PSD95 staining (M1) (E) or anti-PSD95 staining overlapping with PSD95-GFP or GFP-β-actin knock-in (M2) (F) related to Fig 6F–6K. Average values: (PSD95, confocal: median PCC = 0.95, M1 = 0.78, M2 = 0.80, STED: PCC = 0.88, ANOVA, P < 0.001, M1 = 0.62, P < 0.001, M2 = 0.71, P > 0.05, n = 10 neurons) (β-actin, confocal: median PCC = 0.88, P < 0.001, M1 = 0.50, P < 0.001, M2 = 0.74, P > 0.05, STED: median PCC = 0.78, ANOVA, P < 0.001, M1 = 0.18, P < 0.001, M2 = 0.48, P < 0.001, n = 7 neurons). (G and H) gSTED of dendrites and zooms positive for GFP-CaMKIIα knock-in stained with anti-GFP (green, ATTO647N) and anti-PSD95 (magenta, Alexa594). Scale bars, 2 μm and 500 nm for (G) and (H), respectively. (I and J) Line scans of individual spines indicated in (H). Data are represented as means ± SEM. ***P < 0.001. ANOVA. Underlying data can be found in S1 Data. CaMKIIα, Calcium/calmodulin-dependent protein kinase type II subunit alpha; DIV, day in vitro; GFP, green fluorescent protein; gSTED, gated stimulated-emission depletion; ns, not significant; PCC, Pearson’s correlation coefficient; PSD95, postsynaptic density protein 95. (TIF) [file pbio.3000665.s010.tif]

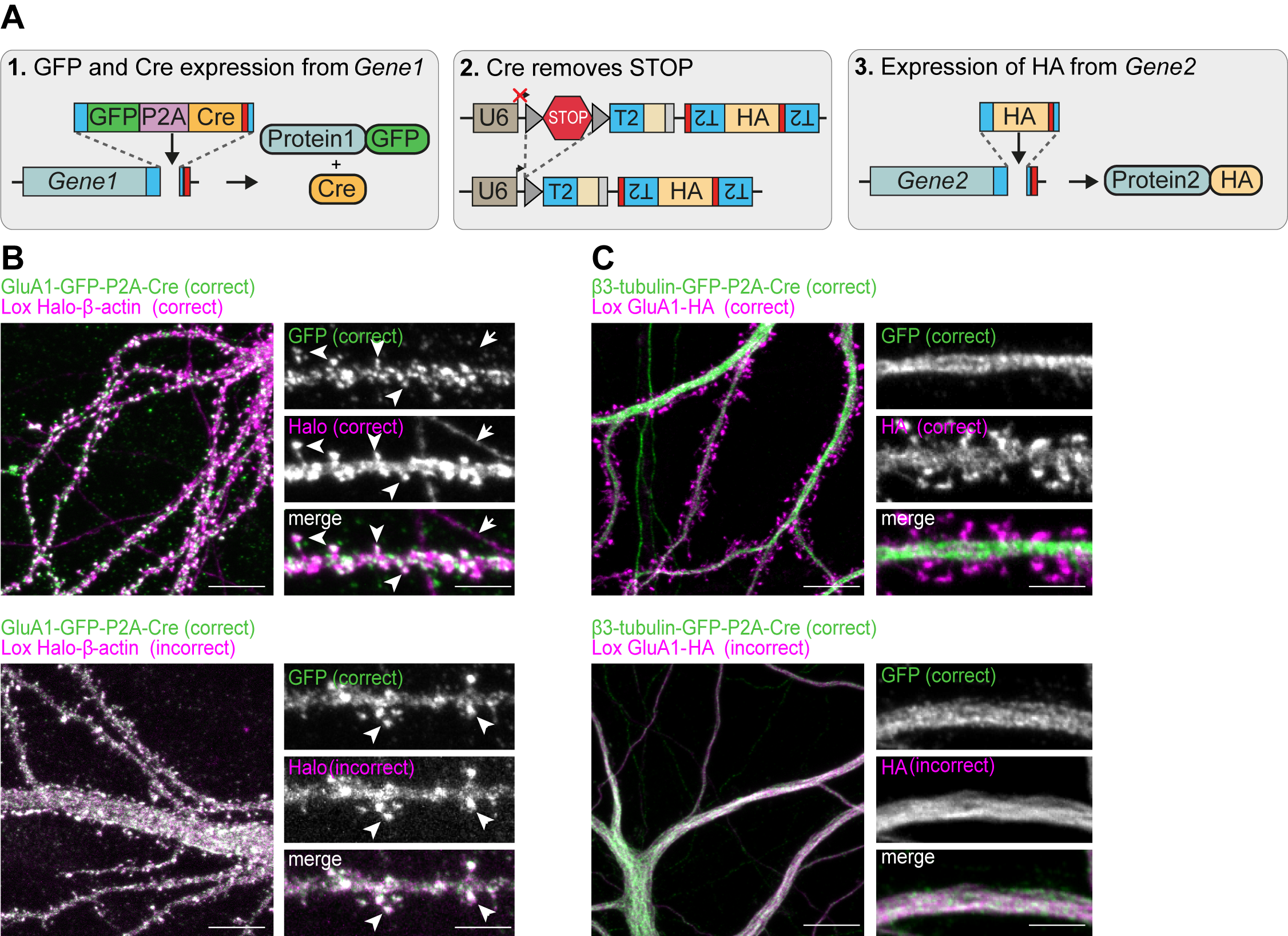

Supplement: S11 Fig — (A) Mechanism of sequential knock-in activation using CAKE. (B) Example of correct dual-color labeling (top) and incorrect dual-color labeling (bottom) with GluA1-GFP-P2A-Cre and Lox Halo β-actin. Arrowheads indicate dendritic spines, and arrow indicates the axon. (C) Example of correct dual-color labeling (top) and incorrect dual-color labeling (bottom) with β3-tubulin-GFP-P2A-Cre and Lox GluA1-HA. Scale bar is 10 μm for overview and 5 μm for zooms. CAKE, conditional activation of knock-in expression; GFP, green fluorescent protein; GluA1, Glutamate receptor AMPA 1; HA, hemagglutinin. (TIF) [file pbio.3000665.s011.tif]
