# Supplementary material for: ORANGE: A CRISPR/Cas9-based genome editing toolbox for epitope tagging of endogenous proteins in neurons
Source: PLoS Biol. 2020 Apr 10;18(4):e3000665. doi: 10.1371/journal.pbio.3000665 (PMC7176289; doi:10.1371/journal.pbio.3000665)
Supplement: S1 Table — ORANGE, Open Resource for the Application of Neuronal Genome Editing. (DOCX) [file pbio.3000665.s012.docx]

| **Gene** | **Protein** | **Endogenous localization** | **Tag location** | **Design rationale** |
| --- | --- | --- | --- | --- |
| *Actb* | β-actin | Cytoskeleton throughout cell, enriched in spines [1] | N-terminal | N-terminal tag is most commonly used, does not interfere with actin localization in neurons, F-actin polymerization or neuronal morphology. See for instance [2], [3], and ﻿[4] |
| *Arpc5* | Arp2/3 complex subunit 5 | Throughout cell, enriched in spines [5] | N-terminal | N-terminal tag does not interfere with Arpc5 localization [3] |
| *Bsn* | Bassoon | Presynaptic active zone [6, 7] | N-terminal | N-terminal GFP tag does not affect localization in neurons [6] |
|  |  |  | C-terminal | C-terminal GFP tag does not affect localization in neurons [6] |
| *Doc2a* | Doc2a | Potentially enriched at synapses [8] | C-terminal | C-terminal GFP tag does not affect function in neurons ([9]) |
| *Cacna1a* | Ca_V_2.1, P/Q type | Presynaptic terminals, dendritic spines [10] | N-terminal | N-terminal tag does not affect protein localization or function [11]. C-terminus contains multiple important localization sequences [12]  and [13] |
| *Cacna1e* | Ca_V_2.3, R type | Around presynaptic terminals, inside dendritic spines and shaft [14] | N-terminal | See *CACNA1A* based on homology. |
| *Cacnb1* | Ca_V_ β1 | Punctate pattern in axons and dendrites, including localization to presynaptic terminals and dendritic spines [15, 16] | N-terminal | Last exon is not included in all splice variants [17] and has few good PAM sites. N-terminal tag does not affect localization or function in homologous proteins [18] |
| *Cacnb2* | Ca_V_ β2 | Punctate pattern in axons and dendrites, including localization to presynaptic terminals and dendritic spines [16] | C-terminal | c-terminal tag does not interfere with localization [16] |
| *Cacnb3* | Ca_V_ β3 | Punctate pattern in axons and dendrites, including localization to presynaptic terminals and dendritic spines [15, 16] | C-terminal | c-terminal tag does not interfere with localization [16] |
| *Cacnb4* | Ca_V_ β4 | Punctate pattern in axons and dendrites, including localization to presynaptic terminals and dendritic spines [15, 16] | C-terminal | c-terminal tag does not interfere with localization [16] |
| *Cacng2* | TARP gamma-2 / Stargazin | Punctate pattern in dendrites, enriched in postsynaptic density [19] | Internal, in C-terminal tail | Internal tag in C-tail does not interfere with localization [20] |
| *Cacng8* | TARP gamma-8 | Punctate pattern in dendrites, enriched in post-synaptic density [19] | Internal, in C-terminal tail | See *Cacng2,* based on homology |
| *Capds* | CAPS1 | Axonal, punctate [21] | N-terminal | Many splice variants lack C-terminus [22] |
| *Camk2a* | CaMKIIα | Soluble throughout cell, moderate enrichment in spines [23] | N-terminal | N-terminal tag does not interfere with localization [24] |
| *Clta* | Clathrin light chain A | Punctate throughout cell [25] | N-terminal | N-terminal tag does not interfere with localization and function [26] |
| *Cplx1* | Complexin1 | Soluble throughout cell [27] | C-terminal | C-terminal GFP overexpression has similar pattern as endogenous [27, 28] |
| *Cplx2* | Complexin2 | Soluble throughout cell [27] | C-terminal | See *Cplx1,* based on homology |
| *Dlg4* | PSD95 | Core component of the postsynaptic density [29] | C-terminal | N-terminal residues important for interaction with membranes. C-terminal tagging is commonly used, see for instance [30], [31], [32], and [33] |
| *Frrs1l* | FRRS1L / C9orf4 | Plasma membrane and ER [34] | C-terminal | N-terminal contains potentially important poly-glycine region. C-terminal tagging did not interfere with localization and co-immunoprecipitation experiments in heterologous cells ﻿[34] |
| *Gria1* | GluA1 | Enriched at the postsynaptic density [35] | C-terminal | Both N and C-terminal tags do not interfere with localization, see for instance [36] and [37] C-terminal tagging was chosen based on availability of PAM sites |
| *Gria2* | GluA2 | Enriched at the postsynaptic density [35] | C-terminal | See *Gria1*, based on homology |
| *Gria3* | GluA3 | Enriched at the postsynaptic density [35] | C-terminal | See *Gria1*, based on homology |
| *Grin1* | GluN1 | Enriched at the postsynaptic density [38] | N-terminal, after signal peptide | C-terminal residues mediate intracellular interactions [39] and some splice variants leak parts of the C-terminal [40] |
| *Grin2a* | GluN2a | Enriched at the postsynaptic density [38] | N-terminal, after signal peptide | C-terminal residues mediate intracellular interactions [39] ﻿ |
| *Grin2b* | GluN2b | Enriched at the postsynaptic density [38] | N-terminal, after signal peptide | C-terminal residues mediate intracellular interactions [39] ﻿ |
| *Gsg1l* | GSG1-l | Punctate pattern in dendrites, enriched at the postsynaptic density [41] | N-terminal | C-terminal tag does not interfere with localization and function [42] |
| *Nlgn3* | Neuroligin-3 | Concentrated in excitatory and inhibitory synapses [43] | N-terminal, after signal peptide | C-terminus mediates intracellular interactions [44] |
| *Pclo* | Piccolo | Enriched at presynaptic active zone [45] | N-terminal | N-terminal domains linked to GFP localize to presynaptic terminals [46]. N-terminus was chosen because PAM sites were predicted to be more specific, based on MIT score |
| *Rab11a* | Rab11 | Punctate labeling of recycling endosomes throughout cell [47, 48] | N-terminal | N-terminal fusion does not affect protein function or localization. See for instance [47, 49] |
| *Rims1* | RIM1 | Enriched at presynaptic active zone [50, 51] | C-terminal | Deletion of C-terminal domains does not interfere with localization [50]. N-terminus contains important interaction sequences [52] |
| *Rims2* | RIM2 | Presynaptic active zone [50, 51] | C-terminal | See *Rims1,* based on homology |
| *Shank1* | Shank1 | Core component of the postsynaptic density [29] | C-terminal | Shanks are commonly tagged at the N-terminus (see e.g. [53], but also [54]), but C-terminal tagging was chosen here due to unavailability of N-terminal PAM sites. We found C-terminal tagging does not interfere with synaptic localization of Shank1 and Shank2 |
| *Shank2* | Shank2 | Core component of the postsynaptic density [29] | C-terminal | See *Shank1*. |
| *Syt7* | Synaptotagmin-7 | Presynaptic terminal [55] , potentially also postsynaptic [56] | N-terminal | Based on homology with *Syt1*: N-terminal tag does not interfere with function, while a C-terminal GFP does interfere with its function in vesicle fusion [57, 58] |
| *Tubb3* | β3-tubulin | Soluble and somatodendritic microtubules [59] | C-terminal | Previously shown to correctly tag *Tubb3* and to be incorporated in the microtubule network. See [60] and [61] |
| *Unc13a* | Munc13-1 | Presynaptic active zone [62] | C-terminal | c-terminal tag does not interfere with localization or function [63] |
| *Wasf1* | WASP1/Wave1 | Cytoplasmic, enriched in spines [64] | C-terminal | C-terminal tag does not interfere with localization [3] |

**Supplemental references**

1. Uemura T, Mori T, Kurihara T, Kawase S, Koike R, Satoga M, et al. Fluorescent protein tagging of endogenous protein in brain neurons using CRISPR/Cas9-mediated knock-in and in utero electroporation techniques. Sci Rep. 2016;6:35861. Epub 2016/10/27. doi: 10.1038/srep35861. PubMed PMID: 27782168; PubMed Central PMCID: PMCPMC5080626.

2. Honkura N, Matsuzaki M, Noguchi J, Ellis-Davies GC, Kasai H. The subspine organization of actin fibers regulates the structure and plasticity of dendritic spines. Neuron. 2008;57(5):719-29. Epub 2008/03/18. doi: 10.1016/j.neuron.2008.01.013. PubMed PMID: 18341992.

3. Chazeau A, Mehidi A, Nair D, Gautier JJ, Leduc C, Chamma I, et al. Nanoscale segregation of actin nucleation and elongation factors determines dendritic spine protrusion. EMBO J. 2014;33(23):2745-64. Epub 2014/10/09. doi: 10.15252/embj.201488837. PubMed PMID: 25293574; PubMed Central PMCID: PMCPMC4282554.

4. Frost NA, Shroff H, Kong H, Betzig E, Blanpied TA. Single-molecule discrimination of discrete perisynaptic and distributed sites of actin filament assembly within dendritic spines. Neuron. 2010;67(1):86-99. Epub 2010/07/14. doi: S0896-6273(10)00422-8 [pii]

10.1016/j.neuron.2010.05.026. PubMed PMID: 20624594; PubMed Central PMCID: PMC2904347.

5. Wegner AM, Nebhan CA, Hu L, Majumdar D, Meier KM, Weaver AM, et al. N-wasp and the arp2/3 complex are critical regulators of actin in the development of dendritic spines and synapses. J Biol Chem. 2008;283(23):15912-20. Epub 2008/04/24. doi: 10.1074/jbc.M801555200. PubMed PMID: 18430734; PubMed Central PMCID: PMCPMC2414292.

6. Dresbach T, Hempelmann A, Spilker C, tom Dieck S, Altrock WD, Zuschratter W, et al. Functional regions of the presynaptic cytomatrix protein bassoon: significance for synaptic targeting and cytomatrix anchoring. Mol Cell Neurosci. 2003;23(2):279-91. Epub 2003/06/19. PubMed PMID: 12812759.

7. Richter K, Langnaese K, Kreutz MR, Olias G, Zhai R, Scheich H, et al. Presynaptic cytomatrix protein bassoon is localized at both excitatory and inhibitory synapses of rat brain. J Comp Neurol. 1999;408(3):437-48. Epub 1999/05/26. doi: 10.1002/(sici)1096-9861(19990607)408:3<437::aid-cne9>3.0.co;2-5. PubMed PMID: 10340516.

8. Courtney NA, Briguglio JS, Bradberry MM, Greer C, Chapman ER. Excitatory and Inhibitory Neurons Utilize Different Ca(2+) Sensors and Sources to Regulate Spontaneous Release. Neuron. 2018;98(5):977-91 e5. Epub 2018/05/15. doi: 10.1016/j.neuron.2018.04.022. PubMed PMID: 29754754; PubMed Central PMCID: PMCPMC6090561.

9. Groffen AJ, Friedrich R, Brian EC, Ashery U, Verhage M. DOC2A and DOC2B are sensors for neuronal activity with unique calcium-dependent and kinetic properties. J Neurochem. 2006;97(3):818-33. Epub 2006/03/07. doi: 10.1111/j.1471-4159.2006.03755.x. PubMed PMID: 16515538.

10. Indriati DW, Kamasawa N, Matsui K, Meredith AL, Watanabe M, Shigemoto R. Quantitative localization of Cav2.1 (P/Q-type) voltage-dependent calcium channels in Purkinje cells: somatodendritic gradient and distinct somatic coclustering with calcium-activated potassium channels. J Neurosci. 2013;33(8):3668-78. Epub 2013/02/22. doi: 10.1523/JNEUROSCI.2921-12.2013. PubMed PMID: 23426693; PubMed Central PMCID: PMCPMC4031662.

11. Schneider R, Hosy E, Kohl J, Klueva J, Choquet D, Thomas U, et al. Mobility of calcium channels in the presynaptic membrane. Neuron. 2015;86(3):672-9. Epub 2015/04/22. doi: 10.1016/j.neuron.2015.03.050. PubMed PMID: 25892305.

12. Kaeser PS, Deng L, Wang Y, Dulubova I, Liu X, Rizo J, et al. RIM proteins tether Ca2+ channels to presynaptic active zones via a direct PDZ-domain interaction. Cell. 2011;144(2):282-95. Epub 2011/01/19. doi: 10.1016/j.cell.2010.12.029. PubMed PMID: 21241895; PubMed Central PMCID: PMCPMC3063406.

13. Lubbert M, Goral RO, Satterfield R, Putzke T, van den Maagdenberg AM, Kamasawa N, et al. A novel region in the CaV2.1 alpha1 subunit C-terminus regulates fast synaptic vesicle fusion and vesicle docking at the mammalian presynaptic active zone. Elife. 2017;6. Epub 2017/08/09. doi: 10.7554/eLife.28412. PubMed PMID: 28786379; PubMed Central PMCID: PMCPMC5548488.

14. Parajuli LK, Nakajima C, Kulik A, Matsui K, Schneider T, Shigemoto R, et al. Quantitative regional and ultrastructural localization of the Ca(v)2.3 subunit of R-type calcium channel in mouse brain. J Neurosci. 2012;32(39):13555-67. Epub 2012/09/28. doi: 10.1523/JNEUROSCI.1142-12.2012. PubMed PMID: 23015445.

15. Ferrandiz-Huertas C, Gil-Minguez M, Lujan R. Regional expression and subcellular localization of the voltage-gated calcium channel beta subunits in the developing mouse brain. J Neurochem. 2012;122(6):1095-107. Epub 2012/06/29. doi: 10.1111/j.1471-4159.2012.07853.x. PubMed PMID: 22737983.

16. Obermair GJ, Schlick B, Di Biase V, Subramanyam P, Gebhart M, Baumgartner S, et al. Reciprocal interactions regulate targeting of calcium channel beta subunits and membrane expression of alpha1 subunits in cultured hippocampal neurons. J Biol Chem. 2010;285(8):5776-91. Epub 2009/12/10. doi: 10.1074/jbc.M109.044271. PubMed PMID: 19996312; PubMed Central PMCID: PMCPMC2820804.

17. Buraei Z, Yang J. The ss subunit of voltage-gated Ca2+ channels. Physiol Rev. 2010;90(4):1461-506. Epub 2010/10/21. doi: 10.1152/physrev.00057.2009. PubMed PMID: 20959621; PubMed Central PMCID: PMCPMC4353500.

18. Wittemann S, Mark MD, Rettig J, Herlitze S. Synaptic localization and presynaptic function of calcium channel beta 4-subunits in cultured hippocampal neurons. J Biol Chem. 2000;275(48):37807-14. Epub 2000/08/10. doi: 10.1074/jbc.M004653200. PubMed PMID: 10931840.

19. Inamura M, Itakura M, Okamoto H, Hoka S, Mizoguchi A, Fukazawa Y, et al. Differential localization and regulation of stargazin-like protein, gamma-8 and stargazin in the plasma membrane of hippocampal and cortical neurons. Neurosci Res. 2006;55(1):45-53. Epub 2006/03/07. doi: 10.1016/j.neures.2006.01.004. PubMed PMID: 16516319.

20. Hafner AS, Penn AC, Grillo-Bosch D, Retailleau N, Poujol C, Philippat A, et al. Lengthening of the Stargazin Cytoplasmic Tail Increases Synaptic Transmission by Promoting Interaction to Deeper Domains of PSD-95. Neuron. 2015;86(2):475-89. Epub 2015/04/07. doi: 10.1016/j.neuron.2015.03.013. PubMed PMID: 25843401.

21. Farina M, van de Bospoort R, He E, Persoon CM, van Weering JR, Broeke JH, et al. CAPS-1 promotes fusion competence of stationary dense-core vesicles in presynaptic terminals of mammalian neurons. Elife. 2015;4. Epub 2015/02/27. doi: 10.7554/eLife.05438. PubMed PMID: 25719439; PubMed Central PMCID: PMCPMC4341531.

22. Nguyen Truong CQ, Nestvogel D, Ratai O, Schirra C, Stevens DR, Brose N, et al. Secretory vesicle priming by CAPS is independent of its SNARE-binding MUN domain. Cell Rep. 2014;9(3):902-9. Epub 2014/12/02. doi: 10.1016/j.celrep.2014.09.050. PubMed PMID: 25437547.

23. Ding JD, Kennedy MB, Weinberg RJ. Subcellular organization of camkii in rat hippocampal pyramidal neurons. J Comp Neurol. 2013;521(15):3570-83. Epub 2013/06/12. doi: 10.1002/cne.23372. PubMed PMID: 23749614; PubMed Central PMCID: PMCPMC4409980.

24. Lu HE, MacGillavry HD, Frost NA, Blanpied TA. Multiple spatial and kinetic subpopulations of CaMKII in spines and dendrites as resolved by single-molecule tracking PALM. J Neurosci. 2014;34(22):7600-10. doi: 10.1523/JNEUROSCI.4364-13.2014. PubMed PMID: 24872564; PubMed Central PMCID: PMC4035521.

25. Blanpied TA, Scott DB, Ehlers MD. Dynamics and regulation of clathrin coats at specialized endocytic zones of dendrites and spines. Neuron. 2002;36(3):435-49. Epub 2002/11/01. PubMed PMID: 12408846.

26. Hoffmann A, Dannhauser PN, Groos S, Hinrichsen L, Curth U, Ungewickell EJ. A comparison of GFP-tagged clathrin light chains with fluorochromated light chains in vivo and in vitro. Traffic. 2010;11(9):1129-40. Epub 2010/06/16. doi: 10.1111/j.1600-0854.2010.01084.x. PubMed PMID: 20545906.

27. Kaeser-Woo YJ, Yang X, Sudhof TC. C-terminal complexin sequence is selectively required for clamping and priming but not for Ca2+ triggering of synaptic exocytosis. J Neurosci. 2012;32(8):2877-85. Epub 2012/02/24. doi: 10.1523/JNEUROSCI.3360-11.2012. PubMed PMID: 22357870; PubMed Central PMCID: PMCPMC3742123.

28. Reim K, Wegmeyer H, Brandstatter JH, Xue M, Rosenmund C, Dresbach T, et al. Structurally and functionally unique complexins at retinal ribbon synapses. J Cell Biol. 2005;169(4):669-80. Epub 2005/05/25. doi: 10.1083/jcb.200502115. PubMed PMID: 15911881; PubMed Central PMCID: PMCPMC2171701.

29. Sheng M, Hoogenraad C. The postsynaptic architecture of excitatory synapses: a more quantitative view. Annu Rev Biochem. 2007;76:823-47.

30. MacGillavry HD, Song Y, Raghavachari S, Blanpied TA. Nanoscale scaffolding domains within the postsynaptic density concentrate synaptic AMPA receptors. Neuron. 2013;78(4):615-22. Epub 2013/05/31. doi: 10.1016/j.neuron.2013.03.009

S0896-6273(13)00256-0 [pii]. PubMed PMID: 23719161.

31. Fortin DA, Tillo SE, Yang G, Rah JC, Melander JB, Bai S, et al. Live imaging of endogenous PSD-95 using ENABLED: a conditional strategy to fluorescently label endogenous proteins. J Neurosci. 2014;34(50):16698-712. Epub 2014/12/17. doi: 10.1523/JNEUROSCI.3888-14.2014. PubMed PMID: 25505322; PubMed Central PMCID: PMCPMC4261096.

32. Sturgill JF, Steiner P, Czervionke BL, Sabatini BL. Distinct domains within PSD-95 mediate synaptic incorporation, stabilization, and activity-dependent trafficking. J Neurosci. 2009;29(41):12845-54. Epub 2009/10/16. doi: 29/41/12845 [pii]

10.1523/JNEUROSCI.1841-09.2009. PubMed PMID: 19828799; PubMed Central PMCID: PMC2787089.

33. Broadhead MJ, Horrocks MH, Zhu F, Muresan L, Benavides-Piccione R, DeFelipe J, et al. PSD95 nanoclusters are postsynaptic building blocks in hippocampus circuits. Sci Rep. 2016;6:24626. doi: 10.1038/srep24626. PubMed PMID: 27109929; PubMed Central PMCID: PMCPMC4842999.

34. Brechet A, Buchert R, Schwenk J, Boudkkazi S, Zolles G, Siquier-Pernet K, et al. AMPA-receptor specific biogenesis complexes control synaptic transmission and intellectual ability. Nat Commun. 2017;8:15910. Epub 2017/07/05. doi: 10.1038/ncomms15910. PubMed PMID: 28675162; PubMed Central PMCID: PMCPMC5500892.

35. Jacob AL, Weinberg RJ. The organization of AMPA receptor subunits at the postsynaptic membrane. Hippocampus. 2015;25(7):798-812. doi: 10.1002/hipo.22404. PubMed PMID: 25524891; PubMed Central PMCID: PMCPMC4472633.

36. Sinnen BL, Bowen AB, Forte JS, Hiester BG, Crosby KC, Gibson ES, et al. Optogenetic Control of Synaptic Composition and Function. Neuron. 2017;93(3):646-60 e5. Epub 2017/01/31. doi: 10.1016/j.neuron.2016.12.037. PubMed PMID: 28132827; PubMed Central PMCID: PMCPMC5300939.

37. Kerr JM, Blanpied TA. Subsynaptic AMPA receptor distribution is acutely regulated by actin-driven reorganization of the postsynaptic density. J Neurosci. 2012;32(2):658-73. Epub 2012/01/13. doi: 10.1523/JNEUROSCI.2927-11.2012. PubMed PMID: 22238102; PubMed Central PMCID: PMCPMC3596168.

38. Sheng M, Kim E. The postsynaptic organization of synapses. Cold Spring Harb Perspect Biol. 2011;3(12). Epub 2011/11/03. doi: 10.1101/cshperspect.a005678. PubMed PMID: 22046028; PubMed Central PMCID: PMCPMC3225953.

39. Kornau HC, Schenker LT, Kennedy MB, Seeburg PH. Domain interaction between NMDA receptor subunits and the postsynaptic density protein PSD-95. Science. 1995;269(5231):1737-40. Epub 1995/09/22. doi: 10.1126/science.7569905. PubMed PMID: 7569905.

40. Hollmann M, Boulter J, Maron C, Beasley L, Sullivan J, Pecht G, et al. Zinc potentiates agonist-induced currents at certain splice variants of the NMDA receptor. Neuron. 1993;10(5):943-54. Epub 1993/05/01. doi: 10.1016/0896-6273(93)90209-a. PubMed PMID: 7684237.

41. Shanks NF, Savas JN, Maruo T, Cais O, Hirao A, Oe S, et al. Differences in AMPA and kainate receptor interactomes facilitate identification of AMPA receptor auxiliary subunit GSG1L. Cell Rep. 2012;1(6):590-8. Epub 2012/07/21. doi: 10.1016/j.celrep.2012.05.004. PubMed PMID: 22813734; PubMed Central PMCID: PMCPMC3401968.

42. Gu X, Mao X, Lussier MP, Hutchison MA, Zhou L, Hamra FK, et al. GSG1L suppresses AMPA receptor-mediated synaptic transmission and uniquely modulates AMPA receptor kinetics in hippocampal neurons. Nat Commun. 2016;7:10873. Epub 2016/03/05. doi: 10.1038/ncomms10873. PubMed PMID: 26932439; PubMed Central PMCID: PMCPMC4778064.

43. Budreck EC, Scheiffele P. Neuroligin-3 is a neuronal adhesion protein at GABAergic and glutamatergic synapses. Eur J Neurosci. 2007;26(7):1738-48. Epub 2007/09/28. doi: 10.1111/j.1460-9568.2007.05842.x. PubMed PMID: 17897391.

44. Shipman SL, Schnell E, Hirai T, Chen BS, Roche KW, Nicoll RA. Functional dependence of neuroligin on a new non-PDZ intracellular domain. Nat Neurosci. 2011;14(6):718-26. Epub 2011/05/03. doi: 10.1038/nn.2825. PubMed PMID: 21532576; PubMed Central PMCID: PMCPMC3171182.

45. Limbach C, Laue MM, Wang X, Hu B, Thiede N, Hultqvist G, et al. Molecular in situ topology of Aczonin/Piccolo and associated proteins at the mammalian neurotransmitter release site. Proc Natl Acad Sci U S A. 2011;108(31):E392-401. Epub 2011/06/30. doi: 10.1073/pnas.1101707108. PubMed PMID: 21712437; PubMed Central PMCID: PMCPMC3150911.

46. Ackermann F, Schink KO, Bruns C, Izsvak Z, Hamra FK, Rosenmund C, et al. Critical role for Piccolo in synaptic vesicle retrieval. Elife. 2019;8. Epub 2019/05/11. doi: 10.7554/eLife.46629. PubMed PMID: 31074746; PubMed Central PMCID: PMCPMC6541439.

47. Mori Y, Matsui T, Furutani Y, Yoshihara Y, Fukuda M. Small GTPase Rab17 regulates dendritic morphogenesis and postsynaptic development of hippocampal neurons. J Biol Chem. 2012;287(12):8963-73. Epub 2012/02/01. doi: 10.1074/jbc.M111.314385. PubMed PMID: 22291024; PubMed Central PMCID: PMCPMC3308742.

48. Sonnichsen B, De Renzis S, Nielsen E, Rietdorf J, Zerial M. Distinct membrane domains on endosomes in the recycling pathway visualized by multicolor imaging of Rab4, Rab5, and Rab11. J Cell Biol. 2000;149(4):901-14. Epub 2000/05/17. doi: 10.1083/jcb.149.4.901. PubMed PMID: 10811830; PubMed Central PMCID: PMCPMC2174575.

49. Hoogenraad CC, Popa I, Futai K, Martinez-Sanchez E, Wulf PS, van Vlijmen T, et al. Neuron specific Rab4 effector GRASP-1 coordinates membrane specialization and maturation of recycling endosomes. PLoS Biol. 2010;8(1):e1000283. Epub 2010/01/26. doi: 10.1371/journal.pbio.1000283. PubMed PMID: 20098723; PubMed Central PMCID: PMCPMC2808209.

50. de Jong APH, Roggero CM, Ho MR, Wong MY, Brautigam CA, Rizo J, et al. RIM C2B Domains Target Presynaptic Active Zone Functions to PIP2-Containing Membranes. Neuron. 2018;98(2):335-49 e7. Epub 2018/04/03. doi: 10.1016/j.neuron.2018.03.011. PubMed PMID: 29606581; PubMed Central PMCID: PMCPMC5910229.

51. Wang Y, Okamoto M, Schmitz F, Hofmann K, Sudhof TC. Rim is a putative Rab3 effector in regulating synaptic-vesicle fusion. Nature. 1997;388(6642):593-8. Epub 1997/08/07. doi: 10.1038/41580. PubMed PMID: 9252191.

52. Lu J, Machius M, Dulubova I, Dai H, Sudhof TC, Tomchick DR, et al. Structural basis for a Munc13-1 homodimer to Munc13-1/RIM heterodimer switch. PLoS Biol. 2006;4(7):e192. Epub 2006/05/31. doi: 10.1371/journal.pbio.0040192. PubMed PMID: 16732694; PubMed Central PMCID: PMCPMC1472246.

53. Sala C, Piech V, Wilson NR, Passafaro M, Liu G, Sheng M. Regulation of dendritic spine morphology and synaptic function by Shank and Homer. Neuron. 2001;31(1):115-30. Epub 2001/08/11. doi: 10.1016/s0896-6273(01)00339-7. PubMed PMID: 11498055.

54. Lilja J, Zacharchenko T, Georgiadou M, Jacquemet G, De Franceschi N, Peuhu E, et al. SHANK proteins limit integrin activation by directly interacting with Rap1 and R-Ras. Nat Cell Biol. 2017;19(4):292-305. Epub 2017/03/07. doi: 10.1038/ncb3487. PubMed PMID: 28263956; PubMed Central PMCID: PMCPMC5386136.

55. Sugita S, Han W, Butz S, Liu X, Fernandez-Chacon R, Lao Y, et al. Synaptotagmin VII as a plasma membrane Ca(2+) sensor in exocytosis. Neuron. 2001;30(2):459-73. Epub 2001/06/08. PubMed PMID: 11395007.

56. Wu D, Bacaj T, Morishita W, Goswami D, Arendt KL, Xu W, et al. Postsynaptic synaptotagmins mediate AMPA receptor exocytosis during LTP. Nature. 2017;544(7650):316-21. Epub 2017/03/30. doi: 10.1038/nature21720. PubMed PMID: 28355182; PubMed Central PMCID: PMCPMC5734942.

57. Han W, Rhee JS, Maximov A, Lin W, Hammer RE, Rosenmund C, et al. C-terminal ECFP fusion impairs synaptotagmin 1 function: crowding out synaptotagmin 1. J Biol Chem. 2005;280(6):5089-100. Epub 2004/11/25. doi: 10.1074/jbc.M408757200. PubMed PMID: 15561725.

58. Yao J, Kwon SE, Gaffaney JD, Dunning FM, Chapman ER. Uncoupling the roles of synaptotagmin I during endo- and exocytosis of synaptic vesicles. Nat Neurosci. 2011;15(2):243-9. Epub 2011/12/27. doi: 10.1038/nn.3013. PubMed PMID: 22197832; PubMed Central PMCID: PMCPMC3435110.

59. Tas RP, Chazeau A, Cloin BMC, Lambers MLA, Hoogenraad CC, Kapitein LC. Differentiation between Oppositely Oriented Microtubules Controls Polarized Neuronal Transport. Neuron. 2017;96(6):1264-71 e5. Epub 2017/12/05. doi: 10.1016/j.neuron.2017.11.018. PubMed PMID: 29198755; PubMed Central PMCID: PMCPMC5746200.

60. Suzuki K, Tsunekawa Y, Hernandez-Benitez R, Wu J, Zhu J, Kim EJ, et al. In vivo genome editing via CRISPR/Cas9 mediated homology-independent targeted integration. Nature. 2016;540(7631):144-9. doi: 10.1038/nature20565. PubMed PMID: 27851729; PubMed Central PMCID: PMCPMC5331785.

61. Parker AL, Teo WS, Pandzic E, Vicente JJ, McCarroll JA, Wordeman L, et al. beta-tubulin carboxy-terminal tails exhibit isotype-specific effects on microtubule dynamics in human gene-edited cells. Life Sci Alliance. 2018;1(2). Epub 2018/08/07. doi: 10.26508/lsa.201800059. PubMed PMID: 30079401; PubMed Central PMCID: PMCPMC6070155.

62. Betz A, Ashery U, Rickmann M, Augustin I, Neher E, Sudhof TC, et al. Munc13-1 is a presynaptic phorbol ester receptor that enhances neurotransmitter release. Neuron. 1998;21(1):123-36. Epub 1998/08/11. PubMed PMID: 9697857.

63. Kalla S, Stern M, Basu J, Varoqueaux F, Reim K, Rosenmund C, et al. Molecular dynamics of a presynaptic active zone protein studied in Munc13-1-enhanced yellow fluorescent protein knock-in mutant mice. J Neurosci. 2006;26(50):13054-66. Epub 2006/12/15. doi: 10.1523/JNEUROSCI.4330-06.2006. PubMed PMID: 17167095; PubMed Central PMCID: PMCPMC6674949.

64. Pilpel Y, Segal M. Rapid WAVE dynamics in dendritic spines of cultured hippocampal neurons is mediated by actin polymerization. J Neurochem. 2005;95(5):1401-10. Epub 2005/09/30. doi: 10.1111/j.1471-4159.2005.03467.x. PubMed PMID: 16190876.
