# Supplementary material for: ORANGE: A CRISPR/Cas9-based genome editing toolbox for epitope tagging of endogenous proteins in neurons
Source: PLoS Biol. 2020 Apr 10;18(4):e3000665. doi: 10.1371/journal.pbio.3000665 (PMC7176289; doi:10.1371/journal.pbio.3000665)
Supplement: S2 Table — ORANGE, Open Resource for the Application of Neuronal Genome Editing. (DOCX) [file pbio.3000665.s013.docx]

| **Gene** | **Protein** | **Target sequence**  **(PAM is underlined)** | **Site of integration**  **(in or before amino acid)** | **MIT score**  **(in rat)** | **Conservation**  **of target sequence**  **in mouse** |
| --- | --- | --- | --- | --- | --- |
| *Actb* | β-actin #1 | CGCAGCGATATCGTCATCCATGG | D2 | 96 | yes |
| *Actb* | β-actin #2 | TGTGCCTTGATAGTTCGCCATGG | 1 bp before ATG | 84 | 1 mismatch |
| *Arpc5* | Arp2/3 complex subunit 5 | CCAGTTCCAGCTGGACTACACGG | STOP | 32 | yes |
| *Bsn* | Bassoon  (N-terminal) | GGGCAACGAGGCCAGCCTGGAGG | L7 | 55 | yes |
| *Bsn* | Bassoon  (C-terminal) | TGGACACAATCACCAGAATGAGG | F3938 | 61 | yes |
| *Cacna1a* | Ca_V_2.1, P/Q | CCGCCTGCGCCGTAGCGGCCCGG | G10 | 93 | yes |
| *Cacna1e* | Ca_V_2.3 R | CAGGATGGCTCGCTTCGGGGAGG | G5 | 88 | yes |
| *Cacnb1* | Ca_v_β1 | CAGAAGAGCGGCATGTCCCGGGG | S8 | 87 | yes |
| *Cacnb2* | Ca_v_β2 | GAGACACGCACGGTCATTGGCGG | Q655 | 85 | 2 mismatches |
| *Cacnb3* | Ca_v_β3 | CAGTAGCTGTCCTTAGGCCAAGG | W479 | 73 | 1 mismatch |
| *Cacnb4* | Ca_v_β4 | GGCAGCCTCAAAGCCTATGTCGG | H417 | 73 | yes |
| *Cacng2* | TARP γ2 | CTTCAACACCCTGCCGTCCACGG | S265 | 86 | yes |
| *Cacng8* | TARP γ8 #1 | GGTGACCGTGACCGTGACGCCGG | V376 | 86 | no |
| *Cacng8* | TARP γ8 #2 | CGTGTTGGTGTTGGACGCGGCGG | A408 | 87 | no |
| *Capds* | CAPS1 | AGTCTCGATCCATAGAGGAAGGG | S39 | 75 | 1 mismatch |
| *Camk2a* | CaMKIIα | CTGCCTGCCCAGTGCCAGGATGG | 2 bp before ATG | 29 | yes |
| *Clta* | Clathrin light chain α | GGATCCAACTCAGCCATGACGG | M1 | 73 | no |
| *Cplx1* | Complexin1 | GTACCTGCCTGGGCCACTGCAGG | L128 | 59 | yes |
| *Cplx2* | Complexin2 | ATATCTGCCGGGGCCACTGCAGG | L128 | 81 | 2 mismatches |
| *Dlg4* | PSD95 | AATCAGAGTCTCTCTCGGGCTGG | R721 | 80 | yes |
| *Doc2a* | Doc2a | CTGCAGTCTGTTCAGGCCAACGG | L402 | 70 | no |
| *Frrs1l* | FRRS1L | tggcttgcagcggaggTTAAGGG | Stop | 70 | 2 mismatches |
| *Gria1* | GluA1 | GGGAGCCACAGGATTGTAACTGG | Stop | 72 | yes |
| *Gria2* | GluA2 | TCGAGAGTGTTAAAATTTAGGGG | Stop | 64 | yes |
| *Gria3* | GluA3 | CAGAAAGTGTTAAGATCTAGGGG | Stop | 71 | yes |
| *Grin1* | GluN1 #1 | TCTTGGGGTCGCAGGCGGCGCGG | A20 | 92 | 1 mismatch |
| *Grin1* | GluN1 #2 | GTTGACGATCTTGGGGTCGCAGG | D23 | 96 | 1 mismatch |
| *Grin1* | GluN1 #3 | CTTGGGGTCGCAGGCGGCGCGGG | A20 | 87 | 1 mismatch |
| *Grin2a* | GluN2a | CGATCCGGCGCAGAACGCGGCGG | A25 | 95 | 2 mismatches |
| *Grin2b* | GluN2b | GACAGCGATGCCGATGCTGGGGGG | S34 | 79 | no |
| *Gsg1l* | GSG1-l | CTGGGGCACTGGGTGTGAtgTGG | Stop | 11 | no |
| *Nlgn3* | Neuroligin-3 | GGTGCTGAGGGCCAGTACCCAGG | T37 | 67 | yes |
| *Pclo* | Piccolo | CAAGCTCGCCTCGTTGCCCATGG | G2 | 61 | yes |
| *Rab11a* | Rab11 | CAGCTCCTCGGCCGCGCCATGGG | 1 bp before ATG | 93 | yes |
| *Rims1* | RIM1 | GGATAGGAGTTTACTATGACCGG | S1615 | 80 | 1 mismatch |
| *Rims2* | RIM2 | TGCTATGAACGAGAGTAAGAAGG | S1551 | 70 | yes |
| *Shank1* | Shank1 | AATTTTTCCTGGAGAGGTGATGG | STOP | 51 | yes |
| *Shank2* | Shank2 | AACAGCTGCTGGACAGATAAGGG | STOP | 32 | yes |
| *Syt7* | Synaptotagmin-7 | GaccatgtaccgggacccggAGG | P5 | 96 | yes |
| *Tubb3* | β3-tubulin | GCTGCGAGCAACTTCACTTGGG | STOP codon | 59 | yes |
| *Unc13a* | Munc13-1 | CAAAACGCGCGCTAGGGCGCAGG | A1762 | 97 | 1 mismatch |
| *Wasf1* | WASP1/Wave1 | GTAGACTGGCTGGAGTGAGAGGG | STOP | 30 | no |
