# Supplementary material for: ORANGE: A CRISPR/Cas9-based genome editing toolbox for epitope tagging of endogenous proteins in neurons
Source: PLoS Biol. 2020 Apr 10;18(4):e3000665. doi: 10.1371/journal.pbio.3000665 (PMC7176289; doi:10.1371/journal.pbio.3000665)
Supplement: S3 Table — (DOCX) [file pbio.3000665.s014.docx]

| **Knock-in** |  | **Primer (5’ 🡪 3’)** |
| --- | --- | --- |
| GFP-β-actin #1 | FW | ATAAAGCTTCGCAGCGATATCGTCATCCAgggctagcgtgagcaagggcgaggag |
|  | RV | ataCTCGAGcccTGGATGACGATATCGCTGCGctgcctgacttgtacagctcgtccatgc |
| GFP-β-actin #2 | FW | tcGAAGACccTAGACCCTGGCGAACTATCAAGGCACATTCGCCATGGCTAGCGGAGTGAGCAAGGGC |
|  | RV | gaGAAGACccCGAGTGTGCCTTGATAGTTCGCCAgGGagcgcttccactCTTGTACAGCTCG |
| Arpc5-GFP | FW | ATAAAGCTTccagttccagctggaCTACAGGGAG GGTAGCGGCTCCAGTAGA |
|  | RV | ATACTCGAGCCCTGTAGtccagctggaactggTTACTTGTACAGCTCGTCCATG |
| GFP-Bassoon | FW | tcGAAGACccTAGACCaCCAGGCTGGCCTCGTTGCCCGCTAGCGGAGTGAGCAAGGGCG |
|  | RV | gaGAAGACccCGAGGGGCAACGAGGCCAGCCTGGaGGcagcgctCTTGTACAGCTCGTCC |
| Bassoon-GFP | FW | tcGAAGACccTAGATGGACACAATCACCAGAATGcGGAgGCTAGCGGAGTGAGCAAGGG |
|  | RV | gaGAAGACccCGAGCCCCATTCTGGTGATTGTGTCCAttaagcgctCTTGTACAGCTCG |
| GFP-Ca_V_2.1, P/Q | FW | ATAAAGCTTCCGCCTGCGCCGTAGCGGCCgggGTGAGCAAGGGCGAGGAG |
|  | RV | atactcgagcccGGCCGCTACGGCGCAGGCGGccccTGAACCCTTGTACAGCTCGTCC |
| GFP-Ca_V_2.3 R | FW | ataaagcttcccCCCCGAAGCGAGCCATCCTGGTGAGCAAGGGCGAGGAG |
|  | RV | atactcgagCAGGATGGCTCGCTTCGGGGgggaaacTGAACCCTTGTACAGCTCGTCC |
| GFP-Ca_v_B1 | FW | ATAAAGCTTCCCCGGGACATGCCGCTCTTCTGgaGCTAGCGGAGTGAGCAAGGGCGAGG |
|  | RV | ATACTCGAGCAGAAGAGCGGCATGTCCCGcGGccagcgctgccCTTGTACAGCTCGTCC |
| Ca_v_B2-GFP | FW | ATAAAGCTTGAGACACGCACGGTCATTGGGGGAGCTAGCGGAGTGAGCAAGGGCGAGGAG |
|  | RV | ATACTCGAGCCCCCAATGACCGTGCGTGTCTCTCAagcgctCTTGTACAGCTCGTCCATG |
| Ca_v_B3-GFP | FW | ATAAAGCTTCAGTAGCTGTCCTTAGGCCAGGGagtGCTAGCGGAGTGAGCAAGGGCGAGG |
|  | RV | ATACTCGAGCCCTGGCCTAAGGACAGCTACTGtcaagcgctCTTGTACAGCTCGTCCATG |
| Ca_v_B4-GFP | FW | ATAAAGCTTGGCAGCCTCAAAGCCTATGTGGGAggtGCTAGCGGAGTGAGCAAGGGCGAG |
|  | RV | ATACTCGAGCCCACATAGGCTTTGAGGCTGCCTCAagcgctCTTGTACAGCTCGTCCATG |
| TARPγ2-GFP | FW | ATAAAGCTTCCCTGGACGGCAGGGTGTTGAAGGTGAGCAAGGGCGAGGAG |
|  | RV | ATACTCGAGCTTCAACACCCTGCCGTCCAGGGtCTTGTACAGCTCGTCCATG |
| TARPγ8-GFP #1 | FW | ATAAAGCTTGGTGACCGTGACCGTGACGCGGGCTAGCGCAGGTAGCGGCTCCAGTAGA |
|  | RV | ATACTCGAGTCCCGCGTCACGGTCACGGTCACCGCagcgctGCCAGCCCCTGCACCCGC |
| TARPγ8-GFP #2 | FW | ATAAAGCTTGCGTGTTGGTGTTGGACGCGGCGGCTAGCGCAGGTAGCGGCTCCAGTAGA |
|  | RV | ATACTCGAGTCCGCCGCGTCCAACACCAACACGCCagcgctGCCAGCCCCTGCACCCGC |
| GFP-CAPS1 | FW | ATAAAGCTTCCCTCGCTGGTGCGGCTTGGAGAGTAGCGGTGGAGTGAGCAAGGGCGAGG |
|  | RV | ATACTCGAGTCTCCAAGCCGCACCAGCGAGGGccTGAACCCTTGTACAGCTCGTCCATG |
| GFP-CaMKIIα | FW | AtaAAGCTTCCCTCCTGGCACTGGGCAGGCAGAGGatggtgagcaagggcgag |
|  | RV | ataCTCGAGCTGCCTGCCCAGTGCCAGGAGGGctgatccCTTGTACAGCTCGTCCATGC |
| mEos3.2-CaMKIIα | FW | AtaTCTAGACCCTCCTGGCACTGGGCAGGCAGGCTAGCACCatgAGTGCGATTAAGCCA |
|  | RV | ataGGATCCCTGCCTGCCCAGTGCCAGGAGGGCTCGAGcTCGTCTGGCATTGTCAGGC |
| GFP-Clathrin LC | FW | ATAAAGCTTGGATCCAACTCAGCCATGAGGGCAGGTAGCGGAgtgagcaagggcgagga |
|  | RV | ATACTCGAGCCCTCATGGCTGAGTTGGATCCTCCGCTACCcttgtacagctcgtccatgc |
| Complexin1-GFP | FW | ATAAAGCTTCCCGCAGTGGCCCAGGCAGGTACAGCGGTGGAGTGAGCAAGGGCGAGG |
|  | RV | ATACTCGAGGTACCTGCCTGGGCCACTGCGGGTCCACCGCTCACTTGTACAGCTCGTCC |
| Complexin2-GFP | FW | ATAAAGCTTCCCGCAGTGGCCCCGGCAGATATGGCTCAGGAGTGAGCAAGGGCGAGG |
|  | RV | ATACTCGAGATATCTGCCGGGGCCACTGCGGGTCCGCCATTACTTGTACAGCTCGTCC |
| PSD95-GFP | FW | ATAAAGCTTAATCAGAGTCTCTCTCGGGCGGGAGCGGTGGAGTGAGCAAGGGCGAGGAG |
|  | RV | ATACTCGAGCCCGCCCGAGAGAGACTCTGATTTTGAACCCTTGTACAGCTCGTCCATG |
| Doc2a-GFP | FW | ATAAAGCTTCTGCAGTCTGTTCAGGCCAAGGGTCTGCTAGCGGAGTGAGCAAGGGCGAGG |
|  | RV | ATACTCGAGCCCTTGGCCTGAACAGACTGCAGTCAagcgctCTTGTACAGCTCGTCC |
| FRRS1L-GFP | FW | ATAAAGCTTtggcttgcagcggaggTTAAGGGAGGTAGCGGCTCCAGTAGA |
|  | RV | ATACTCGAGCCCTTAAcctccgctgcaagccaTTACTTGTACAGCTCGTCCATG |
| GluA1-GFP | FW | ataAAGCTTaCCCGTTACAATCCTGTGGCTCCCGGCGCTAGCGGTAGCGGCTCCAGTAGA |
|  | RV | ataCTCGAGGGGAGCCACAGGATTGTAACGGGAGGCCTTACTTGTACAGCTCGTCCATGC |
| GluA2-GFP | FW | ataAAGCTTCCCCTAAATTTTAACACTCTCGAcTCTGCTAGCGGTAGCGGCTCCAGTAGA |
|  | RV | ataCTCGAGTCGAGAGTGTTAAAATTTAGGGGAGGCCTACTTGTACAGCTCGTCCATGCC |
| GluA3-GFP | FW | ataAAGCTTCCCCTAGATCTTAACACTTTCTGCTCTGCTAGCGGTAGCGGCTCCAGTAGA |
|  | RV | ataCTCGAGCAGAAAGTGTTAAGATCTAGGGGAGGCCTACTTGTACAGCTCGTCCATGCC |
| GFP-GluN1 #1 | FW | ATAAAGCTTTCTTGGGGTCGCAGGCGGCGGGGAGGCAGCGGCGTGAGCAAGGGCGAGGAG |
|  | RV | ATACTCGAGCCCCGCCGCCTGCGACCCCAAGATCCACTGCCCTTGTACAGCTCGTCCATG |
| GFP-GluN1 #2 | FW | ATAAAGCTTGTTGACGATCTTGGGGTCGCAGGCAGCTAGCGGGGTGAGCAAGGGCGA |
|  | RV | ATACTCGAGCCTGCGACCCCAAGATCGTCAACGAAGCGCTTCCCCTAGAGTACAGCTCGT |
| GFP-GluN1 #3 | FW | ATAAAGCTTCTTGGGGTCGCAGGCGGCGCTGGCAGCTAGCGGGGTGAGCAAGGGCGAG |
|  | RV | ATACTCGAGCCAGCGCCGCCTGCGACCCCAAGGAAGCGCTTCCCCTAGAGTACAGCTCGT |
| GFP-GluN2a | FW | ataAAGCTTaCCACCGCGTTCTGCGCCGGATCGCGCGCTAGCGGTAGCGGCTCCAGTAGA |
|  | RV | ataCTCGAGCGATCCGGCGCAGAACGCGGAGGAAGGCCTACTGCCAGCCCCTGCACCCGC |
| GFP-GluN2b | FW | ataAAGCTTGACAGCGATGCCGATGCTGGGAGGTCAGCTAGCGGTAGCGGCTCCAGTAGA |
|  | RV | ataCTCGAGCCTCCCAGCATCGGCATCGCTGTCAGGCCTACTAGCCCCTGCACCCGCTCC |
| GSG1-l-GFP | FW | ATAAAGCTTCCCcaTCACACCCAGTGCCCCAGAAGGTAGCGGCTCCAGTAGA |
|  | RV | ATACTCGAGCTGGGGCACTGGGTGTGAtgGGGTTACTTGTACAGCTCGTCCATG |
| Nlgn3-GFP | FW | ATAAAGCTTCCCGGGTACTGGCCCTCAGCACCATGGTGAGCAAGGGCGAGGAG |
|  | RV | ATACTCGAGGGTGCTGAGGGCCAGTACCCGGGtCTTGTACAGCTCGTCCATG |
| Piccolo-GFP | FW | tcGAAGACccTAGACAAGCTCGCCTCGTTGCCCAcGGAgGCTAGCGGAGTGAGCAAGGGC |
|  | RV | gaGAAGACccCGAGCCCTGGGCAACGAGGCGAGCTTGccagcgctCTTGTACAGCTCGTC |
| GFP-Rab11 | FW | ATAAAGCTTCCCATggcgcggccgaggagctgAATGGTGAGCAAGGGCGAGGAG |
|  | RV | ATACTCGAGcagctcctcggccgcgccATGGGttCTTGTACAGCTCGTCCATG |
| RIM1-GFP | FW | ataAAGCTTGGATAGGAGTTTACTATGACGGGATCTGCTAGCGGTAGCGGCTCCAGTAGA |
|  | RV | ataCTCGAGCCCGTCATAGTAAACTCCTATCCTAGGCCTACTTGTACAGCTCGTCCATGC |
| RIM2-GFP | FW | ATAAAGCTTTGCTATGAACGAGAGTAAGAGGGtctGCTAGCGGAGTGAGCAAGGGCGAGG |
|  | RV | ATACTCGAGCCCTCTTACTCTCGTTCATAGCAtcaagcgctCTTGTACAGCTCGTCCATG |
| Shank1-GFP | FW | ATAAAGCTTCCCTCACCTCTCCAGGAAAAATTAGTGAGCAAGGGCGAGGAG |
|  | RV | ATACTCGAGAATTTTTCCTGGAGAGGTGAGGGTTACTTGTACAGCTCGTCCATG |
| Shank2-GFP | FW | ATAAAGCTTCCCTTATCTGTCCAGCAGCTGTTAGTGAGCAAGGGCGAGGAG |
|  | RV | ATACTCGAGAACAGCTGCTGGACAGATAAGGGTTACTTGTACAGCTCGTCCATG |
| GFP-Syt7 | FW | ATAAAGCTTCCCccgggtcccggtacatggtcGCTAGCGGAGTGAGCAAGGGCGAGGAGc |
|  | RV | ATACTCGAGgaccatgtaccgggacccggcGGaagcgctCTTGTACAGCTCGTCCATGcc |
| β3-tubulin-GFP | FW | ataAAGCTTGCTGCGAGCAACTTCACTTGGGggatcaggcgtgagcaagggcgaggag |
|  | RV | ataCTCGAGCCCAAGTGAAGTTGCTCGCAGCacattacttgtacagctcgtccatgc |
| Munc13-1-GFP | FW | ataaagcttCAAAACGCGCGCTAGGGCGCGGGAGCGGTGGAGTGAGCAAGGGCGAGGAG |
|  | RV | ATACTCGAGCCCGCGCCCTAGCGCGCGTTTTGTTACTTGTACAGCTCGTCCATG |
| WASP1-GFP | FW | ATAAAGCTTCCCtcTCACTCCAGCCAGTCTACAAGGTAGCGGCTCCAGTAGA |
|  | RV | ATACTCGAGGTAGACTGGCTGGAGTGAgaGGGTTACTTGTACAGCTCGTCCATG |
