# Supplementary material for: ORANGE: A CRISPR/Cas9-based genome editing toolbox for epitope tagging of endogenous proteins in neurons
Source: PLoS Biol. 2020 Apr 10;18(4):e3000665. doi: 10.1371/journal.pbio.3000665 (PMC7176289; doi:10.1371/journal.pbio.3000665)
Supplement: S4 Table — (DOCX) [file pbio.3000665.s015.docx]

| **Gene** | **Knock-in** | **Junction** | **Forward (5’ 🡪 3’)** | **Reverse (5’ 🡪 3’)** | **Size product (bp)** |
| --- | --- | --- | --- | --- | --- |
| *Actb* | GFP-β-actin #1 | 5’ | TTCCTTTGTCCCCTGAGCTT | CTGAACTTGTGGCCGTTTAC | 237 |
|  |  | 3’ | CATGGTCCTGCTGGAGTTCGTG | ATACCCACCATCACACCCTG | 312 |
| *Arpc5* | Arpc5-GFP | 5’ | TGCCTGGGGCATAATAGGTG | CTGAACTTGTGGCCGTTTAC | 283 |
|  |  | 3’ | CATGGTCCTGCTGGAGTTCGTG | GCTGAGAGAAACAGATGCTACC | 192 |
| *Cacna1a* | GFP-Ca_V_2.1 | 5’ | TCTTTGCAGAATGGCCCG | CTGAACTTGTGGCCGTTTAC | 129 |
| *Cacna1e* | GFP-Ca_V_2.3 | 5’ | TGTGTGGATAAGGCTCCGA | CTGAACTTGTGGCCGTTTAC | 217 |
|  |  | 3’ | CATGGTCCTGCTGGAGTTCGTG | TTTGACTGCTTGTAGGCGGC | 198 |
| *Cacng2* | TARPγ2-GFP | 3’ | CATGGTCCTGCTGGAGTTCGTG | AGCTGTTATCCCTGTCCGAGT | 164 |
| *Cadps* | GFP-CAPS1 | 5’ | CATCTTTTGCACCCCAAGCC | CTGAACTTGTGGCCGTTTAC | 283 |
| *Camka2a* | GFP-CaMKIIα | 3’ | CATGGTCCTGCTGGAGTTCGTG | CAAGAGGCTGATCCCCATGC | 190 |
| *Cplx1* | Complexin1-GFP | 5’ | CGACCCAAGAAGGCTATCCC | CTGAACTTGTGGCCGTTTAC | 206 |
|  |  | 3’ | CATGGTCCTGCTGGAGTTCGTG | GTCAGTGATGGCAGTACGGG | 302 |
| *Dlg4* | PSD95-GFP | 5’ | TGACTTGCAGCCATCGTAGA | CTGAACTTGTGGCCGTTTAC | 200 |
|  |  | 3’ | CATGGTCCTGCTGGAGTTCGTG | TGGGCTATTCAGTCCACACCA | 175 |
| *Frrs1l* | FRRS1L-GFP | 5’ | TCAGAGCGCGTTGTCAGTAT | CTGAACTTGTGGCCGTTTAC | 248 |
|  |  | 3’ | CATGGTCCTGCTGGAGTTCGTG | TGCCTTGACAACCCTGTCTGA | 264 |
| *Gria1* | GluA1-GFP | 5’ | CAAGTCCATGCAATCCATTCCC | CTGAACTTGTGGCCGTTTAC | 201 |
|  |  |  | CATGGTCCTGCTGGAGTTCGTG | TGTCCTCCATATGGTCGTGGT | 204 |
| *Gria2* | GluA2-GFP | 3’ | CATGGTCCTGCTGGAGTTCGTG | AGGACGCGACAAGAAAAGCTAA | 144 |
| *Gria3* | GluA3-GFP | 5’ | CCACCAACACTCAGAATTACGC | CTGAACTTGTGGCCGTTTAC | 205 |
|  |  | 3’ | CATGGTCCTGCTGGAGTTCGTG | CACTGACAAGAGAGCACGGTC | 241 |
| *Grin1* | GFP-GluN1 #1 | 5’ | CCCGGGGCTCCTAGAGAA | CTGAACTTGTGGCCGTTTAC | 276 |
|  |  | 3’ | CATGGTCCTGCTGGAGTTCGTG | CCAAGAGCCGTGTCGCTTAT | 199 |
| *Grin2a* | GFP-GluN2a | 5’ | TGGGGTCGGGTTTCATACTTG | CTGAACTTGTGGCCGTTTAC | 335 |
|  |  | 3’ | CATGGTCCTGCTGGAGTTCGTG | AGTTCGCGTTCTGTCACGTC | 195 |
| *Grin2b* | GFP-GluN2b | 5’ | AGGATGTGTTCCACAACGTG | CTGAACTTGTGGCCGTTTAC | 297 |
|  |  | 3’ | CATGGTCCTGCTGGAGTTCGTG | CACCCGGGGAACTACTGAGA | 229 |
| *Gsg1l* | GSG1-l-GFP | 5’ | AAGCAGCAGAGCTAAACCGT | CTGAACTTGTGGCCGTTTAC | 175 |
|  |  | 3’ | CATGGTCCTGCTGGAGTTCGTG | GAGCTGAGTCCACAGCACAG | 165 |
| *Nlgn3* | Neuroligin-3-GFP | 5’ | CCTATTGGGCTGATGCTGTGA | CTGAACTTGTGGCCGTTTAC | 274 |
|  |  | 3’ | CATGGTCCTGCTGGAGTTCGTG | TACCCCCAGGTATTGGTCCAC | 178 |
| *Rab11a* | GFP-Rab11 | 5’ | AAGCTCTTCGCTCGGGTTAC | CTGAACTTGTGGCCGTTTAC | 196 |
|  |  | 3’ | CATGGTCCTGCTGGAGTTCGTG | CCCCAAATGGGCTATCGGTG | 147 |
| *Rims1* | RIM1-GFP | 5’ | GGGTGTGGCTCAAATCTTGTT | CTGAACTTGTGGCCGTTTAC | 291 |
|  |  | 3’ | CATGGTCCTGCTGGAGTTCGTG | GTGGCTCTCACTAGCATGTCC | 316 |
| *Rims2* | RIM2-GFP | 5’ | GGCCGCATGGATCACAAATC | CTGAACTTGTGGCCGTTTAC | 284 |
|  |  | 3’ | CATGGTCCTGCTGGAGTTCGTG | TTATCCCTAGACGTGGGCTCC | 227 |
| *Shank1* | Shank1-GFP | 5’ | ACCACGAGATTGATGGCTCC | CTGAACTTGTGGCCGTTTAC | 223 |
|  |  | 3’ | CATGGTCCTGCTGGAGTTCGTG | GGGGCAGGACACAGTTTTAGC | 209 |
| *Shank2* | Shank2-GFP | 5’ | ACAATGAGATTGACGGCAGC | CTGAACTTGTGGCCGTTTAC | 223 |
|  |  | 3’ | CATGGTCCTGCTGGAGTTCGTG | TGCTTGGCCACCACACATTT | 156 |
| *Syt7* | GFP-Syt7 | 5’ | AGCGGCGGCAGAGAAG | CTGAACTTGTGGCCGTTTAC | 194 |
| *Tubb3* | β3-tubulin-GFP | 5’ | CCGAGAGCAACATGAACGAC | CTGAACTTGTGGCCGTTTAC | 217 |
|  |  | 3’ | CATGGTCCTGCTGGAGTTCGTG | AAAGGTGGCTAAAACGGGGAG | 312 |
| *Unc13a* | Munc13-1-GFP | 5’ | TCAAGCTCAAGTCCGACACG | CTGAACTTGTGGCCGTTTAC | 151 |
|  |  | 3’ | CATGGTCCTGCTGGAGTTCGTG | ATCCCGCCCTGTATTTTGCAG | 321 |
| *Wasf1* | WASP1-GFP | 5’ | AGCGGATTGAAAACGACGTG | CTGAACTTGTGGCCGTTTAC | 232 |
|  |  | 3’ | CATGGTCCTGCTGGAGTTCGTG | AACATTTTCAAAGATCAAGCGCCA | 203 |
