# Supplementary material for: ORANGE: A CRISPR/Cas9-based genome editing toolbox for epitope tagging of endogenous proteins in neurons
Source: PLoS Biol. 2020 Apr 10;18(4):e3000665. doi: 10.1371/journal.pbio.3000665 (PMC7176289; doi:10.1371/journal.pbio.3000665)
Supplement: S5 Table — ORANGE, Open Resource for the Application of Neuronal Genome Editing. (DOCX) [file pbio.3000665.s016.docx]

| **Gene** | **Protein** | **Plasmid name** | **Addgene ID** |
| --- | --- | --- | --- |
| *Actb* | β-actin (KI #1) | pORANGE GFP-Actb KI | 131479 |
|  | β-actin (KI #2) | pORANGE GFP-Actb KI #2 | 139666 |
| *Arpc5* | Arp2/3 complex subunit 5 | pORANGE GFP-Arpc5 KI | 131503 |
| *Bsn* | Bassoon (N-terminal) | pORANGE GFP-Bsn KI | 139664 |
|  | Bassoon (C-terminal) | pORANGE Bsn-GFP KI | 139665 |
| *FRRS1L* | Frrs1l (C9orf4) | pORANGE C9orf4-GFP KI | 131472 |
| *Cacna1a* | Ca_V_2.1, (P/Q type) | pORANGE GFP-CACNA1A KI | 131480 |
| *Cacna1e* | Ca_V_2.3 (R type) | pORANGE GFP-CACNA1E KI | 131481 |
| *Cacnb1* | Ca_v_ β1 | pORANGE GFP-Cacnb1 KI | 139660 |
| *Cacnb2* | Ca_v_ β2 | pORANGE Cacnb2-GFP KI | 139661 |
| *Cacnb3* | Ca_v_ β3 | pORANGE Cacnb3-GFP KI | 139662 |
| *Cacnb4* | Ca_v_ β4 | pORANGE Cacnb4-GFP KI | 139663 |
| *Cacng2* | TARP γ2 | pORANGE CACNG2-GFP KI | 131504 |
| *Cacng8* | TARP γ8 (KI #1) | pORANGE CACNG8-GFP KI #1 | 131473 |
|  | TARP γ8 (KI #2) | pORANGE CACNG8-GFP KI #2 | 131474 |
| *Cadps* | CAPS1 | pORANGE GFP-CADPS KI | 131482 |
| *Camk2a* | CaMKIIα | pORANGE GFP-Camk2a KI | 131484 |
|  |  | pORANGE mEos3.2-Camk2a KI | 131493 |
| *Ctla* | Clathrin light chain α | pORANGE GFP-Clta KI | 131483 |
| *Cplx1* | Complexin1 | pORANGE Cplx1-GFP KI | 131475 |
| *Cplx2* | Complexin2 | pORANGE Cplx2-GFP KI | 131476 |
| *Dlg4* | PSD95 | pORANGE Dlg4-GFP KI | 131477 |
| *Doc2a* | Doc2a | pORANGE Doc2A-GFP KI | 131478 |
| *Gria1* | GluA1 | pORANGE Gria1-GFP KI | 131489 |
| *Gria2* | GluA2 | pORANGE Gria2-GFP KI | 131490 |
| *Gria3* | GluA3 | pORANGE Gria3-GFP KI | 131491 |
| *Grin1* | GluN1 #1 | pORANGE GFP-Grin1 KI | 131485 |
|  | GluN1 #2 | pORANGE GFP-Grin1 KI #2 | 139658 |
|  | GluN1 #3 | pORANGE GFP-Grin1 KI #3 | 139659 |
| *Grin2a* | GluN2a | pORANGE GFP-Grin2a KI | 131486 |
| *Grin2b* | GluN2b | pORANGE GFP- Grin2b KI | 131487 |
| *GSG1-l* | GSG1-l | pORANGE GSG1l-GFP KI | 131492 |
| *Nlgn3* | Neuroligin-3 | pORANGE GFP-Nlgn3 KI | 131501 |
| *Pclo* | Piccolo | pORANGE GFP-Pclo KI | 139657 |
| *Rab11a* | Rab11 | pORANGE GFP-RAB11a KI | 131499 |
| *Rims1* | RIM1 | pORANGE Rims1-GFP KI | 131494 |
| *Rims2* | RIM2 | pORANGE Rims2-GFP KI | 131495 |
| *Shank1* | Shank1 | pORANGE Shank1-GFP KI | 131500 |
| *Shank2* | Shank2 | pORANGE Shank2-GFP KI | 131496 |
| *Syt7* | Synaptotagmin-7 | pORANGE GFP-Syt7 KI | 131488 |
| *Tubb3* | β3-tubulin | pORANGE Tubb3-GFP KI | 131497 |
| *Unc13a* | Munc13-1 | pORANGE unc13a-GFP KI | 131498 |
| *WASF* | WASP1/Wave1 | pORANGE WASF1-GFP KI | 131502 |
| *Lentiviral constructs* | | pFUGW-mCherry-KASH | 131505 |
|  |  | pFUGW SpCas9 | 131506 |
|  |  | pFUGW ORANGE Gria1-GFP KI _ mCherry-KASH | 131507 |
|  |  | pFUGW ORANGE Tubb3-GFP KI _ mCherry-KASH | 131508 |
|  |  | pFSW FLEx mCherry-KASH | 139652 |
|  |  | pFSW FLEx Synapsin-FLAG | 139653 |
| *AAV constructs* | | pAAV MCS mCherry KASH | 139654 |
|  |  | pAAV ORANGE Gria1a HaloTag KI | 139655 |
|  |  | pAAV ORANGE Dlg4 HaloTag KI | 139656 |
| *Cloning template vector* | | pORANGE Cloning template vector | 131471 |
|  |  | pORANGE LOX Cloning template vector | 139651 |
