# Supplementary material for: ORANGE: A CRISPR/Cas9-based genome editing toolbox for epitope tagging of endogenous proteins in neurons
Source: PLoS Biol. 2020 Apr 10;18(4):e3000665. doi: 10.1371/journal.pbio.3000665 (PMC7176289; doi:10.1371/journal.pbio.3000665)
Supplement: Attachment — Submitted filename: Willems et al - Response to reviewers.docx [file pbio.3000665.s019.docx]

First and foremost, we appreciate the very constructive comments of all reviewers. Based on the reviews, we have made major improvements on the manuscript, including two new main figures and five new supplemental figures. We also updated most other figures.

First, we put particular emphasis on the validity of the ORANGE toolbox, using next-generation sequencing of 28 knock-ins, and quantification of immunocytochemistry to assess protein levels. We also made major improvements on the performance of several knock-in constructs. Second, we added multiple datasets demonstrating application of ORANGE tools, including live imaging, *in vivo* genome editing, usage of multiple epitope tags, and multiplex editing for visualization of multiple proteins.

Taken together, we feel that our new results significantly strengthen the main conclusions of our manuscript and underscore the potential of ORANGE for neurobiological research. Below, we address all comments from the reviewers point-by-point. At the end we included a list of references that was added during the revision.

**Reviewer #1**

In general, the authors established a very good tagging toolkit for monitoring the endogenous protein by using HITI-CRISPR techniques for taking the advantages of HITI can work with higher efficiency in post-mitotic cells. With the combination of the high resolution of gSTED microscopy images, ORANGE Toolkit indeed provides a powerful approach to monitor the expression and dynamics in living cells, especially for those proteins without good antibodies.

We would like to thank the reviewer for these very positive comments and recognizing the potential of our toolbox.

There are some minor comments regarding this manuscript:

1. For HITI-CRISPR precise KI analysis, it is strongly suggested that only analyze the eGFP-positive cells instead of analyze a pool of cells. A N-tag of eGFP might cause out-of-frame for the coding sequences of target gene, when the other allele is also edited, a KO cell was generated. If the target region is not analyzed in sorted cells (i.e. eGFP positive cells), the aberrant expression pattern cannot be ruled out unless a specific antibody was used parallely to double check the pattern.

We agree with the reviewer that in some neurons, a partial or full knock-out was created as shown in Figure 3 (now Figure 4). As also raised by reviewer #4, this is important information, especially in the context of neuronal networks, or when pools of cells are analyzed. Importantly, we found that most knock-in positive neurons showed expression at endogenous levels (Figure 4 and S8).

To extend our observations, we have validated protein levels for several more proteins including Shank2 (C-terminal), CaMKIIα (N-terminal) (Figure S8) and Bassoon (both N- and C-terminal, Figure 4, see lines 266-290). In addition, we have further analyzed indel frequencies for a large set of proteins (28) using next generation sequencing (Figure S6, lines 204-216) Also, we have now optimized the efficiency and fidelity of several knock-in constructs by testing and comparing several PAM sites within the same gene (Figure S7, line 217-228), giving important leads for the design of future constructs. Together, these experiments provide in-depth insight into the efficiency and accuracy of the knock-in constructs.

Finally, we like to add that in most experiments we used lipofection to deliver the knock-in plasmid. Since lipofection only delivers plasmids to about 1% of all cells on a coverslip, >90% of all neurons will remain unaffected. Thus, while ORANGE knock-ins may lead to reduction of endogenous levels in a small population of cells, the overwhelming majority of cells in the culture are unaffected, and thus any potential effects at the neuronal network level will be negligible.

2. In Figure S3-D, due to duplication of incomplete target sequences (17 bases, 5’tggcactgggcaggcaggg) in the 5’juntion in GFP-CamKIIa, a new ATG start codon was generated. This new ATG-frame will cause an out-of-frame reading to the subsequent eGFP coding sequence. To overcome this situation, extra bases must have been added to the primer when constructing the donor plasmid. And this kind of minor sequence modification must be target-specific. If no extra bases were added, the so-called precise KI cannot result in correct eGFP expression. If extra bases were added, the authors must have mentioned it in the text to remind those who are interested in using ORAGNE in the future.

Indeed, for this knock-in we target the gene before the original start-codon. In addition to the ATG present before the GFP tag itself, an additional ATG motif is introduced by the incomplete target sequence. Extra bases were introduced such that, even if this additional ATG is used as a start codon, it will be in-frame with the GFP coding sequence. We have included a detailed description of this approach in the methods section. (lines 926-928)

3. From the previous study of Palmer et al., (Comp Funct Genomics. 2004 Jun; 5(4): 342–353.doi: 10.1002/cfg.405), N-terminal tagging with GFP adversely affects the protein localization in reverse transfection assays, whereas tagging with GFP at the C-terminal is generally better in preserving the localization of the native protein. To further confirmation of the correct expression of eGFP-tagged protein, especially for those with N-tagged (i.e. CADPS, Syt7, CACNA1A, CACNA1E, Grin1, Grin2a and Grin2b in table S1), a specific antibody must be used. The authors should put the evidences for the co-localization of endogenous detected by eGFP and specific antibody, not just mention in the discussion.

Tagging proteins with GFP can always adversely affect protein localization and/or function but has been used extensively in many different model systems in the past decades without adverse effects. We have based the choice of where to tag the proteins carefully on the available literature where possible. While indeed generally C-terminal tags are used for the majority of proteins, for multiple of the proteins of our interest, N-terminal tagging is preferred for several reasons. For instance, *CACNA1A* and *CACNA1E* contain important targeting sequences at the C-terminus (Kaeser *et al.,* Cell 2011, Lubbert *et al.*, eLife 2017), and N-terminal GFP tags do not adversely affect channel localization or function (Schneider *et al.,* 2017). Additionally, for some membrane proteins, N-terminal tagging allows live-cell detection of surface-expressed proteins, and has been shown to not affect trafficking, subcellular distribution and function (see for instance: Barria *et al.* Neuron 2002 and Kopec *et al.* J Neurosci 2006 for N-terminal tagging of NMDA receptor subunits). We made similar, rational decisions for all other targeted genes based on literature, or sequence information.

The reviewer asks specifically for additional antibody staining for several specific proteins, however, for many of the knock-ins, specific, commercially available antibodies suitable for immunohistochemistry are lacking. This was, in fact, one of the main motivations for us to develop a CRISPR-based knock-in approach, to visualize proteins for which no high-quality tools exist in literature. We added several alternative lines of evidence to the manuscript to address the reviewer’s question regarding N-terminally tagged proteins.

First, we added a table listing the used resources and literature on which our choice for the tag insertion site were made, as well as the known endogenous localization described in literature (Table S1). Second, we compared the localization of several N- and C-terminally tagged proteins to the localization of endogenous synaptic proteins (Figure S3, line 158-163). Third, we made several additional N-terminal knock-ins for β-actin and GluN1, in which a different PAM site, and thus tag insertion site, was used. These knock-ins show identical subcellular localization, implying that tag location has no effect on protein localization (Figure S7, line 217-228). Fourth, we developed constructs for Bassoon targeting both the N- and C-terminus, and both knock-ins show identical subcellular localization (Figure S3, line 159-161). Additionally, since for this protein a specific antibody is available, we confirmed correct localization of both Bassoon knock-in constructs using immunostaining (Figure 4, line 266-290). Finally, the active zone protein Piccolo is known to closely co-localize with Bassoon. We created an N-terminal GFP knock-in for Piccolo, which closely colocalized with antibody stainings for Bassoon (Figure S3).

Taken together, we agree with the reviewer that tag location can affect protein distribution and should be chosen carefully, but we designed knock-ins in our library to negate these effects as much as possible. Our new experimental data further demonstrate that N-terminally knock-ins in our library are consistent with endogenous localization, based on immunocytochemistry and existing literature.

**Reviewer #2**

The authors present a systematic approach of on-locus tagging of neuronal proteins (cultivated hippocampal neurons, slice culture) based on adaptations of CRISPR/Cas9 technology (they name their approach ORANGE). They present successful on locus tagging, and subject the tagged proteins to conventional co-labelling experiments, as well super-resolution and single molecule imaging experiments.

Let me start with the notion that I am not an expert in the application of CRISPR/Cas9 technology to mammalian neurons. I do feel that their approach is relatively standard but might well be wrong.

We agree with the reviewer that CRISPR/Cas9 technology has been around for several years now and has also been used for genome editing in mammalian neurons in a few proof-of-principle studies, but the presented knock-in technology is far from being a standard approach in most molecular neurobiology labs. Our approach builds on these studies and presents a versatile system that we demonstrate can be easily expanded to a large number of proteins, including less abundant but essential synaptic proteins, and can be combined with super-resolution imaging approaches to yield new biological insights. We provide multiple improvements over existing methods: e.g. extensive validation using quantitative immunostainings (Figure 4, S8) and next generation sequencing (Figure S6), exchangeable tags (Figure 1), and multiplex genome editing Figure 8), and we describe essential steps to design and optimize well performing knock-ins (Figures S1, S5, S7). We strongly believe that this toolbox and guidelines will dramatically expand the use of CRISPR/Cas9 technology in neuroscience and related fields in cell biology. We would like to highlight that our pre-print manuscript received considerable attention and we have already distributed our ORANGE toolbox to over 25 laboratories, indicating the strong interest from the scientific community for ORANGE.

Concerning the subsequent characterization on chosen examples: this is as far as I can see done largely well, however, certainly pertains only to their chosen examples. Quite honestly, I do not think that stating that endogenously labelled neuronal proteins can per se suffice to retrieve super-resolved or single molecule data (here this least certainly) is particularly surprising. There are quite a few examples of this kind, also from non-mammalian neurobiology.

We did not intend to suggest “that endogenously labelled neuronal proteins can per se suffice to retrieve super-resolved or single molecule data”. We do provide a general workflow for how tagging endogenous proteins can facilitate super-resolution investigation of protein distribution. We also give a few examples on how this method can be used, for instance for NMDA receptors, for which suitable tools are currently lacking, hampering progress in the field.

Overall, my enthusiasm for this study is rather moderate as it really seems a rather standard approach. Again, in case my colleagues are of different opinion concerning the novelty and importance of this “resource” this reviewer would not object an overall more positive judgement.

Specific points:

-	Ensuring XFP-on locus integration certainly is not a per se guarantee for functionality, as the sheer presence of the XFP can certainly be problematic. This from my experience is particularly relevant for membrane proteins such as glutamate receptors. Thus: ultimately to generation of such a resource would need strategies to functionally validate the fusion products. This, at least in my view, must be done by genetic rescue experiments targeting specific phenotypes of the gene under study. This, however, can hardly be part of such a resource approach.

There is no doubt that integration of fluorescent tags can affect localization and/or functionality of the protein of interest, see also our response to Reviewer #1. We have been very careful to choose the optimal site of integration, building on decades of experience in the literature with tagging (neuronal) proteins (summarized in Table S1). Additionally, the fluorophore location for most knock-ins were previously validated by many labs in overexpression studies. In particular, for glutamate receptors, tagging has been validated extensively by numerous labs and has been shown to not affect synthesis, trafficking, anchoring, and channel functionality (see e.g. Barria *et al.* Neuron 2002 and Kopec *et al.* J Neurosci 2006). In the revised manuscript, we included a table with literature for every knock-in construct used to choose the optimal tag insertion site (Table S1).

-	Along similar lines: tagging exclusively N- or C-termini will not suffice ultimately to arrive a fully functional versions, particularly for membrane proteins.

Indeed, we agree that tagging a protein can always intrinsically alter its functionality. Nevertheless, we have carefully designed each of the knock-in strategies and we included Table S1, documenting these decisions and comparison with known subcellular localization based on overexpression, subcellular fractionation and immunocytochemistry. Additionally, we added a new data figure, demonstrating correct synaptic targeting of multiple knock-in fusion proteins, both for presynaptic active zone proteins as well as components of the postsynaptic density (Figure S3, lines 158-163).

-	They honestly state: “besides correct integration, we found various insertions and deletions leading to frameshift mutations. We noted that the frequency of indels was highly variable between different knock-ins, which is likely because the accuracy of Cas9 and NHEJ is reported to highly depend on the target sequence (25, 26)”:

but isnt that potentially dangerous, as e.g. the second allele of a targeted locus could be become unfunctional or worse start to express gain-of-function partial proteins etc? What is the exact frequency of such events compared to “correct” ones?

This is a valid point and therefore, we performed next-generation sequencing analysis on 28 proteins. This analysis showed that for most genes, indel frequencies are relatively low, with most knock-ins showing more than 50% correct integration (Figure S6, lines 204-216). This suggests that for these genes, indels are a relatively minor concern. For some knock-ins, however, we did detect a very high indel rate. For instance, for *Actb* (β-actin) and *Grin1* (GluN1), we observed correct integration for less than 10% of the reads. This corresponds well with our experience using these knock-ins, for instance in terms of number of knock-ins cells per culture. Based on these results, we decided to design novel knock-ins for these genes, targeting alternative PAM sites around the original locus. Strikingly, we found that alternative gRNA sequences can lead to up to 10-fold improvement of knock-in efficiency (Figure S7, line 217-228). Thus, while indels do occur, it can be largely circumvented by optimizing knock-in design. We carefully address this in the results (line 217-228) and discussion (line 479-505) sections of the revised manuscript. We thank the reviewer for raising this issue, as we feel that these experiments significantly improved our manuscript.

-	“While this fatefully labels proteins of interest”: Do they really mean that?

We have rephrased this (line 87).

-	Why is there in Fig. 3E no GFP group?

In figure 3E (now Figure 4E), we specifically aimed to compare the enrichment of GFP-tagged PSD95 in spines of knock-in cells and overexpression based on the GFP signal and therefore, the GFP fill is not quantified here.

**Reviewer #3**

Genome editing through CRISPR/Cas9 has revolutionised biology in recent years. Many laboratories have developed CRISPR/Cas9 based techniques to insert Tag/ORF DNA sequences with great precision at any desired genomic locus in both dividing and postmitotic cells. These technologies are improving rapidly and have proven invaluable in addressing a wide range of questions in research fields such as cancer biology, developmental biology and neuroscience.

Willems and colleagues offer an original contribution to this expanding repertoire of genome editing tools. They have developed a simple cassette, which they dubbed pORANGE (Open Resource for the Application of Neuronal Genome Editing), that, through a simple two-step cloning procedure can specify any gene/protein for in frame tagging with a GFP. The authors note that the GFP can easily be replaced with any other fluorescent protein or any other tag, greatly expanding the range of experimental approaches one could choose. Further important advantages of the method include: 1) The expression at physiological levels of the tagged proteins as opposed to most traditional overexpression methods. 2) Less likely to interfere with protein localisation and function. 3) Compatible with super resolution of tagged proteins of live as well as fixed tissues.

Additionally, the authors have generated an extensive library of ‘target’ plasmids that allows tagging with GFP of a large number of proteins of interest to researchers interested in synapse biology.

The potential and versatility of the tools are excellently illustrated using the PSD95 and NMDA receptor GFP tagging in primary neurons and hippocampal slice cultures. The authors show that the approach is compatible with different gene delivery systems. Super resolution microscopy, live cell imaging and single molecule localisation microscopy are used to illustrate the proper behaviour of EGF-tagged NMDA receptors. Quantification of fluorescence intensities demonstrate the tagged proteins are expressed at normal physiological level, greatly adding confidence to this approach.

It is envisaged that these tools can easily be adapted for molecules of interest in other aspects of the neurosciences and other fields of cell biological research and as such should appeal to a broad scientific audience.

The authors should indicate how interested colleagues can obtain the plasmids. Will they be deposited with Addgene?

This is very positive feedback, and we very much appreciate highlighting these aspects of our study. At this moment, most plasmids are already available via Addgene. We added an overview with Addgene numbers (Table S5). Constructs that were developed during the revision are currently being submitted to Addgene, and we plan to add all Addgene references before publication of the manuscript.

**Reviewer #4**

In this manuscript, Willems et al. present an interesting strategy and toolkit for genome engineering in order to label endogenous neuronal proteins for optical imaging. The strategy is interesting and offers potential as an alternative to overexpression of fluorescently-tagged proteins. A particularly interesting aspect of this paper is the development of a construct library for a series of target proteins which could represent a useful resource for the community. However, the manuscript, as presented, contains a number of pitfalls and limitations that should be addressed, as well as certain demonstrations that fall short of showing the usefulness of their approach.

1- A major concern with this approach is the presence of indels and frameshift mutations in many cells in which GFP is not expressed. The authors downplay the impact of the partial or complete knockout of the target protein in non fluorescent cells, but this could impact the network signaling, thereby reducing the applicability of this approach. Furthermore, the outcome of the second copy of the gene in GFP positive cells remains unclear. The author suggest that expression levels of PSD95 was “modestly lower” in some GFP positive cells. Could the downregulation be more pronounced for other constructs (no data was shown for this)? If so, then the level of expression of the targeted protein could impact synaptic, cytoskeletal (etc) function. Or could some be mutated ? If so, this will impact function of multimeric proteins.

We are aware of this concern and did not intend to downplay this. In fact, we addressed this thoroughly and presented the data openly in our manuscript (Figure 3, now Figure 4 (updated), Figure S8 (new), and Figure S3 (replaced by Figure S6). Also, it is important to note that in most of the experiments, only an insignificant fraction of the cells (<1%) in the network is targeted by the transfection method we used (lipofection), ruling out any significant effects on network signaling. We now mention these considerations more explicitly in the text (lines 483-485) and have included validation of additional knock-in constructs for which antibodies are available (Figure 4H, I and Figure S8, lines 266-290). Furthermore, we performed an in-depth analysis of indel frequency with next-generation sequencing on a large number of knock-ins from our library (Figure S6) (lines 204-216). For most knock-ins, we detected correct integration in more than 50% of all reads, and for some knock-ins this was over 90%. For several knock-ins that showed a high indel frequency, we designed new knock-in constructs, with up to 10-fold increase in performance (Figure S7) (lines 217-228). Taken together, while we agree that indels introduced by ORANGE may be a concern under some specific experimental conditions, we feel that in most situations the indel frequency is sufficiently low that this does not affect experimental outcome. We have included all points above in the updated version of the manuscript.

2- The applicability of ORANGE would be most relevant in live-cell experiments, without using antibodies or nanobodies. However, the authors show only one live-cell experiment relying on the signal of a transfected protein is a supplementary figure. In fact, while the authors argue in the introduction of fixation and immunolabeling can introduce ultrastructural changes and interfere with protein localization, for the most part, their study uses fixation and/or immunolabeling.

We included a new data figure in the manuscript (Figure 5) with two new live-cell experiments (lines 292-311). These include time-lapse imaging of GFP-β-actin, as well as FRAP analysis of GFP-β-actin in individual dendritic spines. Together with single-molecule tracking of mEos3.2-CaMKIIα (Figure S9) and nanobody based uPAINT for GFP-GluN1 (Figure 7), we feel that this sufficiently demonstrates that ORANGE knock-ins can be used for live imaging. We also have rephrased the statements made in the introduction (lines 53-60).

3- On lines 147-149 the authors state “For none of the knock-in constructs in our library, we observed aberrant or diffuse expression of the integrated tag, indicating that off-target integration, or unintended GFP expression directly from the knock-in plasmid did not occur or is extremely rare”.

How was aberrant expression assessed for each protein tested (other than PSD95, b3-tubulin and b-actin) ?

It would be useful to describe any aberrant or diffuse expression for other tested constructs that were not included in this library and if this is more susceptible to happen with particular types of proteins.

We have assessed the distribution of all the knock-in constructs we generated by evaluating multiple coverslips with neurons expressing each construct. This means we sampled multiple GFP+ cells per knock-in (>100 cells for some constructs). We compared the staining pattern from these results with available literature which we summarized in Table S1 (new) with references to the literature we used. Furthermore, we generated several new knock-in constructs for β-actin and GluN1 targeting different sequences (Figure S7) (lines 217-228), as well as for Bassoon (Figure 4H, I), all showing the same distribution pattern. We have never observed knock-in constructs that yield aberrant or unexpected distribution patterns. We have included this point more clearly in the text (lines 199-203 and 494-520) and included an additional data figure in which several knock-ins were co-stained with synaptic markers (Figure S3) (lines 158-163).

Importantly, there are several mechanism that prevent erroneous expression of GFP with ORANGE knock-ins (also see discussion, lines 499-505):

1. We selected gRNA sequences with high MIT scores (usually >50, preferentially >75). As a result, the gRNA sequence itself is unique within the genome, and the most likely off targets are intergenic, or within introns.
2. In case the donor is still integrated within a genomic coding sequence, the mechanism for direction selectivity (Figure 1B, S1) will most likely not work, meaning that 50% of the off-target integration will be in the inverted orientation.
3. All our donor DNAs are designed for the reading frame of the target locus. If this donor DNA is integrated at an off-target locus, only 1 in 3 of these loci will be in the correct frame. This will reduce erroneous expression of GFP by a further 66%.

Considering our observations with our complete library, we feel that well-designed knock-ins do not negatively affect localization of the labeled protein in neurons.

4- Throughout the paper, figures mostly show single cell compartments. It would be useful to show large fields of view to appreciate the level and localization of expression in multiple cells.

For most of our experiments, we used lipofection-based methods for DNA delivery, targeting <1% of all neurons in a culture resulting in sparse labeling of cells, limiting the chance of finding labeled neurons close to each other. In contrast, *in vivo* labeling using AAV, results in a higher density of labeled cells. For both, we have included several lower magnifications examples of knock-in cells in culture and tissue (Figures 1, 2, and 3).

5- While the authors tested several proteins that are yet uncharacterized (e.g. c9orf4 and GSG1L), they did not show their cellular distribution using super-resolution. All of the super-resolution experiments were focussed on previously observed proteins, thus reducing the novelty impact of their study.

Indeed, we used PSD95 and NDMARs, proteins that have been extensively characterized, to establish a firm benchmark for our approach but we agree that ORANGE is well suited to study proteins that are less well characterized. We took up the suggestion of the reviewer and acquired gSTED images of FRRS1L/C9orf4 and GSGL1 (Figure 6L-N) (lines 361-375). We found that whereas GSG1L partially localized in dendritic spines, FRRS1L was mostly located in the dendritic shaft, closely associated with the endoplasmic reticulum as suggested by previous studies (also see discussion lines 532-542).

6- For the super-resolution imaging experiments, the GFP signal was amplified with either nanobodies or antibodies against GFP. This is not always clearly stated in the text and should be standardized throughout the manuscript.

Indeed, for the presented super-resolution approaches, labeling with appropriate fluorophores is required for dSTORM (single-molecule blinking of the Alexa647 dye), gSTED (higher resolution with organic dyes like Alexa488, Alexa594 and ATTO647N), and for live-cell single-molecule tracking (nanobody-conjugated to ATTO647N). We have stated this more clearly in the legends by mentioning when enhancement with anti-GFP was used. We also added the name of the dye that was used (if any).

7- In line 339, it is stated that receptor trajectories were “found within the boundaries of the PSD”. This should be rephrased to avoid confusion with PSD95 staining that was used throughout the study, since in this case, as described in the figure legend (Fig7F), the “PSD-mask” was obtained from overexpressed Homer1c-mCherry widefield signal. For a better representation of the synaptic areas, the authors could have used PSD95-FingR co-transfection.

We chose Homer1c-mCherry as a synaptic marker as we, and other labs, found that it reliably marks synaptic sites without affecting synaptic morphology or function (see for instance Scheefhals *et al.,* Cell Reports 2019). In our experience, PSD95-FingR labeled with red fluorescent proteins such mCherry tends to aggregate. We have adjusted the wording in the manuscript to avoid confusion between PSD (now referred to as synapse) and PSD95 (the protein) throughout the manuscript.

8- Multicolor imaging experiments presented throughout the paper exploit the ORANGE strategy for only one protein, while the other is revealed from either immunostaining or overexpression of fluorescently tagged proteins. Could the ORANGE strategy be applicable to multicolor labeling ? As multicolor imaging is nowadays essential in almost every experimental protocol, they should demonstrate how this can be achieved with the ORANGE strategy.

We fully agree that multiplex imaging would be a very useful application. However, creating double knock-ins in individual neurons, has been a major challenge in the field. Existing methods have quite low efficiency (Mikuni *et al.* Cell 2016, using HDR), or are limited to smaller tags and a high potential rate of knockout alleles (Gao *et.al.* Neuron 2019, using NHEJ). The challenge with NHEJ-based approaches like ORANGE, is that when to loci are edited simultaneously, the donor DNA has no preference in which locus it is integrated. Therefore, there is no control over which fluorophore is fused to which protein.

To overcome this, we devised a mechanism of genetic sequential integration of donor DNAs with two knock-in plasmids. The first knock-in contains a donor DNA sequence coding for GFP-P2A-Cre, to create a GFP fusion protein and bi-cistronic expression of Cre recombinase. The second knock-in contains a Lox-STOP-Lox cassette in the U6 promoter, preventing expression of the gRNA, unless Cre removes the Lox-STOP-Lox cassette. Thus, the second knock-in is only activated after the first knock-in is completed, and functional proteins have been produced from the edited allele.

Using this approach, we successfully created double knock-ins for a variety of genes (Figure 8, lines: results, 442-458 and discussion, 559-575). We feel that although this approach still requires further optimization, our experiments convincingly demonstrate that this strategy for generating double knock-ins is a considerably important addition to the ORANGE toolbox facilitating the localization of multiple endogenous proteins simultaneously in individual cells.

**Added references:**

Dong JX, Lee Y, Kirmiz M, Palacio S, Dumitras C, Moreno CM, et al. A toolbox of nanobodies developed and validated for use as intrabodies and nanoscale immunolabels in mammalian brain neurons. Elife. 2019;8. Epub 2019/10/01. doi: 10.7554/eLife.48750. PubMed PMID: 31566565; PubMed Central PMCID: PMCPMC6785268.

Matsuda T, Oinuma I. Imaging endogenous synaptic proteins in primary neurons at single-cell resolution using CRISPR/Cas9. Mol Biol Cell. 2019;30(22):2838-55. Epub 2019/09/12. doi: 10.1091/mbc.E19-04-0223. PubMed PMID: 31509485; PubMed Central PMCID: PMCPMC6789158.

Platt RJ, Chen S, Zhou Y, Yim MJ, Swiech L, Kempton HR, et al. CRISPR-Cas9 knockin mice for genome editing and cancer modeling. Cell. 2014;159(2):440-55. Epub 2014/09/30. doi: 10.1016/j.cell.2014.09.014. PubMed PMID: 25263330; PubMed Central PMCID: PMCPMC4265475.

Bae S, Kweon J, Kim HS, Kim JS. Microhomology-based choice of Cas9 nuclease target sites. Nat Methods. 2014;11(7):705-6. Epub 2014/06/28. doi: 10.1038/nmeth.3015. PubMed PMID: 24972169.

Dresbach T, Hempelmann A, Spilker C, tom Dieck S, Altrock WD, Zuschratter W, et al. Functional regions of the presynaptic cytomatrix protein bassoon: significance for synaptic targeting and cytomatrix anchoring. Mol Cell Neurosci. 2003;23(2):279-91. Epub 2003/06/19. PubMed PMID: 12812759.

Hotulainen P, Llano O, Smirnov S, Tanhuanpaa K, Faix J, Rivera C, et al. Defining mechanisms of actin polymerization and depolymerization during dendritic spine morphogenesis. J Cell Biol. 2009;185(2):323-39. Epub 2009/04/22. doi: 10.1083/jcb.200809046. PubMed PMID: 19380880; PubMed Central PMCID: PMCPMC2700375.

Korobova F, Svitkina T. Molecular architecture of synaptic actin cytoskeleton in hippocampal neurons reveals a mechanism of dendritic spine morphogenesis. Mol Biol Cell. 2010;21(1):165-76. Epub

Zheng CY, Petralia RS, Wang YX, Kachar B, Wenthold RJ. SAP102 is a highly mobile MAGUK in spines. J Neurosci. 2010;30(13):4757-66. Epub 2010/04/02. doi: 10.1523/JNEUROSCI.6108-09.2010. PubMed PMID: 20357126; PubMed Central PMCID: PMCPMC2874826.2009/11/06. doi: 10.1091/mbc.E09-07-0596. PubMed PMID: 19889835; PubMed Central PMCID: PMCPMC2801710.

Bissen D, Foss F, Acker-Palmer A. AMPA receptors and their minions: auxiliary proteins in AMPA receptor trafficking. Cell Mol Life Sci. 2019;76(11):2133-69. Epub 2019/04/03. doi: 10.1007/s00018-019-03068-7. PubMed PMID: 30937469; PubMed Central PMCID: PMCPMC6502786.

Gu X, Mao X, Lussier MP, Hutchison MA, Zhou L, Hamra FK, et al. GSG1L suppresses AMPA receptor-mediated synaptic transmission and uniquely modulates AMPA receptor kinetics in hippocampal neurons. Nat Commun. 2016;7:10873. Epub 2016/03/05. doi: 10.1038/ncomms10873. PubMed PMID: 26932439; PubMed Central PMCID: PMCPMC4778064.

Schwenk J, Boudkkazi S, Kocylowski MK, Brechet A, Zolles G, Bus T, et al. An ER Assembly Line of AMPA-Receptors Controls Excitatory Neurotransmission and Its Plasticity. Neuron. 2019;104(4):680-92 e9. Epub 2019/10/13. doi: 10.1016/j.neuron.2019.08.033. PubMed PMID: 31604597.

Brechet A, Buchert R, Schwenk J, Boudkkazi S, Zolles G, Siquier-Pernet K, et al. AMPA-receptor specific biogenesis complexes control synaptic transmission and intellectual ability. Nat Commun. 2017;8:15910. Epub 2017/07/05. doi: 10.1038/ncomms15910. PubMed PMID: 28675162; PubMed Central PMCID: PMCPMC5500892.

Han W, Wang H, Li J, Zhang S, Lu W. Ferric Chelate Reductase 1 Like Protein (FRRS1L) Associates with Dynein Vesicles and Regulates Glutamatergic Synaptic Transmission. Front Mol Neurosci. 2017;10:402. Epub 2017/12/26. doi: 10.3389/fnmol.2017.00402. PubMed PMID: 29276473; PubMed Central PMCID: PMCPMC5727121.

Back S, Necarsulmer J, Whitaker LR, Coke LM, Koivula P, Heathward EJ, et al. Neuron-Specific Genome Modification in the Adult Rat Brain Using CRISPR-Cas9 Transgenic Rats. Neuron. 2019;102(1):105-19 e8. Epub 2019/02/23. doi: 10.1016/j.neuron.2019.01.035. PubMed PMID: 30792150.

Lindeboom RG, Supek F, Lehner B. The rules and impact of nonsense-mediated mRNA decay in human cancers. Nat Genet. 2016;48(10):1112-8. Epub 2016/09/13. doi: 10.1038/ng.3664. PubMed PMID: 27618451; PubMed Central PMCID: PMCPMC5045715

Lin JY, Knutsen PM, Muller A, Kleinfeld D, Tsien RY. ReaChR: a red-shifted variant of channelrhodopsin enables deep transcranial optogenetic excitation. Nat Neurosci. 2013;16(10):1499-508. Epub 2013/09/03. doi: 10.1038/nn.3502. PubMed PMID: 23995068; PubMed Central PMCID: PMCPMC3793847.

Verhaagen J, Hobo B, Ehlert EME, Eggers R, Korecka JA, Hoyng SA, et al. Small Scale Production of Recombinant Adeno-Associated Viral Vectors for Gene Delivery to the Nervous System. Methods Mol Biol. 2018;1715:3-17. Epub 2017/12/01. doi: 10.1007/978-1-4939-7522-8_1. PubMed PMID: 29188502.

Grimm D, Kay MA, Kleinschmidt JA. Helper virus-free, optically controllable, and two-plasmid-based production of adeno-associated virus vectors of serotypes 1 to 6. Mol Ther. 2003;7(6):839-50. Epub 2003/06/06. doi: 10.1016/s1525-0016(03)00095-9. PubMed PMID: 12788658.

Connelly JP, Pruett-Miller SM. CRIS.py: A Versatile and High-throughput Analysis Program for CRISPR-based Genome Editing. Sci Rep. 2019;9(1):4194. Epub 2019/03/14. doi: 10.1038/s41598-019-40896-w. PubMed PMID: 30862905; PubMed Central PMCID: PMCPMC6414496.

de Jong APH, Roggero CM, Ho MR, Wong MY, Brautigam CA, Rizo J, et al. RIM C2B Domains Target Presynaptic Active Zone Functions to PIP2-Containing Membranes. Neuron. 2018;98(2):335-49 e7. Epub 2018/04/03. doi: 10.1016/j.neuron.2018.03.011. PubMed PMID: 29606581; PubMed Central PMCID: PMCPMC5910229

Lindhout FW, Cao Y, Kevenaar JT, Bodzeta A, Stucchi R, Boumpoutsari MM, et al. VAP-SCRN1 interaction regulates dynamic endoplasmic reticulum remodeling and presynaptic function. EMBO J. 2019;38(20):e101345. Epub 2019/08/24. doi: 10.15252/embj.2018101345. PubMed PMID: 31441084; PubMed Central PMCID: PMCPMC6792018.
