# Supplementary material for: ORANGE: A CRISPR/Cas9-based genome editing toolbox for epitope tagging of endogenous proteins in neurons
Source: PLoS Biol. 2020 Apr 10;18(4):e3000665. doi: 10.1371/journal.pbio.3000665 (PMC7176289; doi:10.1371/journal.pbio.3000665)
Supplement: Attachment — Submitted filename: Willems et al - Response to reviewers.docx [file pbio.3000665.s020.docx]

We thank the reviewers for their time and careful evaluation of our revised manuscript.
